# MITF and c-Jun antagonism interconnects melanoma dedifferentiation with pro-inflammatory cytokine responsiveness and myeloid cell recruitment

**DOI:** 10.1038/ncomms9755

**Published:** 2015-11-04

**Authors:** Stefanie Riesenberg, Angela Groetchen, Robert Siddaway, Tobias Bald, Julia Reinhardt, Denise Smorra, Judith Kohlmeyer, Marcel Renn, Bengt Phung, Pia Aymans, Tobias Schmidt, Veit Hornung, Irwin Davidson, Colin R. Goding, Göran Jönsson, Jennifer Landsberg, Thomas Tüting, Michael Hölzel

**Affiliations:** 1 Unit for RNA Biology, Institute for Clinical Chemistry and Clinical Pharmacology, University Hospital Bonn, Sigmund-Freud-Strasse 25, 53105 Bonn, Germany; 2 Ludwig Institute for Cancer Research, Nuffield Department of Clinical Medicine, University of Oxford, Old Road Campus Research Building, Headington, Oxford OX3 7DQ, UK; 3 Laboratory for Experimental Dermatology, Department of Dermatology, University Hospital Bonn, Sigmund-Freud-Strasse 25, 53105 Bonn, Germany; 4 Division of Oncology and Pathology, Department of Clinical Sciences, Lund University, Barngatan 2B, Lund 221 85, Sweden; 5 Institute of Molecular Medicine, University Hospital, University of Bonn, Sigmund-Freud-Strasse 25, 53127 Bonn, Germany; 6 Department of Functional Genomics and Cancer, Institut de Génétique et de Biologie Moléculaire et Cellulaire, CNRS/INSERM/ULP, 1 Rue Laurent Fries, Illkirch Cédex 67404, France

## Abstract

Inflammation promotes phenotypic plasticity in melanoma, a source of non-genetic heterogeneity, but the molecular framework is poorly understood. Here we use functional genomic approaches and identify a reciprocal antagonism between the melanocyte lineage transcription factor MITF and c-Jun, which interconnects inflammation-induced dedifferentiation with pro-inflammatory cytokine responsiveness of melanoma cells favouring myeloid cell recruitment. We show that pro-inflammatory cytokines such as TNF-α instigate gradual suppression of MITF expression through c-Jun. MITF itself binds to the c-Jun regulatory genomic region and its reduction increases c-Jun expression that in turn amplifies TNF-stimulated cytokine expression with further MITF suppression. This feed-forward mechanism turns poor peak-like transcriptional responses to TNF-α into progressive and persistent cytokine and chemokine induction. Consistently, inflammatory MITF^low^/c-Jun^high^ syngeneic mouse melanomas recruit myeloid immune cells into the tumour microenvironment as recapitulated by their human counterparts. Our study suggests myeloid cell-directed therapies may be useful for MITF^low^/c-Jun^high^ melanomas to counteract their growth-promoting and immunosuppressive functions.

## Abstract (web summary)

The c-Jun transcription factor can mediate a cell's response to TNFa. Here, Riesenberg *et al*. show in melanoma cells that c-Jun has an inverse relationship with the key melanocyte transcription factor MITF and that high c-Jun levels contribute to melanoma heterogeneity and an inflammatory microenvironment.

## Graphical abstract

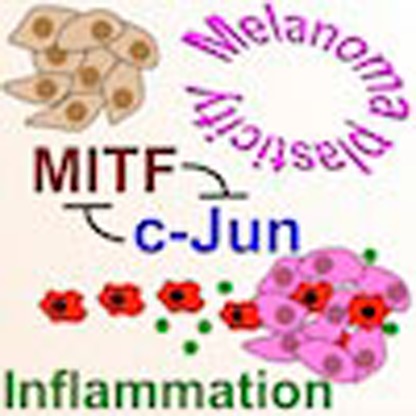

Malignant melanoma is an aggressive cancer that originates from the pigment producing melanocytes in the skin^1^. Early metastatic spread has been linked to its neural crest origin, a transient, highly migratory and multipotent embryonic cell population that gives rise to diverse cell lineages including Schwann cells, peripheral neurons and melanocytes^2^. Phenotypic plasticity is an essential property of the neural crest to respond to morphogenetic cues from the tissue microenvironment and to initiate the respective lineage programmes in a proper temporospatial manner^3^. These developmental traits provide an explanation for the aggressive behaviour of neural crest-derived tumours such as melanoma and it emphasizes the need to dissect the molecular mechanisms controlling phenotypic plasticity^4^,^5^.

We previously showed that reciprocal interactions between melanoma and immune cells in a pro-inflammatory microenvironment provide a source of phenotypic heterogeneity that drives therapy resistance and metastasis^4^,^6^. Using a genetically engineered mouse model we found that an effective immunotherapy with adoptively transferred T cells (pmel-1 T cells) directed against the melanocytic target antigen gp100 (also known as Pmel) caused regressions of established melanomas but tumours invariably recurred. Unexpectedly, late relapse melanomas exhibited a global loss of melanocytic differentiation markers and a vice versa upregulation of the neural-crest progenitor marker NGFR. In that study, we identified a cascade of changes in the tumour microenvironment that were responsible for this phenotype switch. Melanoma-infiltrating cytotoxic T cells elicited an extensive inflammatory response that subsequently triggered the recruitment of myeloid immune cells. Released pro-inflammatory cytokines such tumour necrosis factor (TNF)-α induced dedifferentiation of the melanoma cells and thereby suppressed the expression of the melanocytic target antigen gp100. This abrogated recognition and killing by the cytotoxic pmel-1 T cells and favoured the outgrowth of melanomas with a dedifferentiated NGFR^+^ phenotype. Hence, inflammatory signals emerged as crucial instigators of phenotypic plasticity in melanoma causing heterogeneity beyond the diversity of the genomic aberrations^7^.

In the past years, several studies have demonstrated that human melanoma cells appear in distinct cell states also called ‘proliferative' and ‘invasive'^8^,^9^. At the heart of this concept, the ‘phenotype switching model', lies the melanocytic lineage transcription factor MITF (microphthalmia-associated transcription factor) and opposing EMT (epithelial–mesenchymal transition)-like and hypoxia-related programmes^10^,^11^,^12^,^13^,^14^,^15^,^16^,^17^. MITF functions as a potent ‘rheostat' that dictates the phenotypic appearance of melanoma cells^18^,^19^. Intermediate levels of MITF strongly support melanoma cell growth, whereas both increased and reduced levels cause cell cycle arrest either by differentiation or a senescence-like response^18^,^19^,^20^. Intriguingly, a series of studies identified phenotype switches linked to MITF induction or repression in the context of resistance to BRAF inhibitors in both cell lines and melanoma patient samples^21^,^22^,^23^,^24^. This highlights the importance of identifying the molecular mechanisms driving phenotypic plasticity, as this would provide new opportunities for phenotype-directed therapies counteracting BRAF inhibitor resistance. We focus on inflammation as a source of phenotypic diversity and the interactions of melanoma and immune cells, because we hypothesize that melanoma cell states actively determine the immune cell composition of the tumour microenvironment in a reciprocal manner with important implications for melanoma immunotherapies^6^,^7^. Therefore, we are particularly interested in the poorly understood molecular mechanisms that orchestrate inflammation-induced phenotype switches of melanoma cells.

Here we identify an antagonism between MITF and c-Jun as a molecular interface between pro-inflammatory signals from the tumour microenvironment and melanoma cell plasticity. The transcription factor c-Jun is known to synergize with nuclear factor-κB (NF-κB) in the transcriptional response to pro-inflammatory cytokines such as TNF-α and to amplify TNF-stimulated cytokine expression. We found that MITF and c-Jun transcriptionally repress each other and therefore c-Jun induction by TNF-α instigates a feed-forward loop of melanoma dedifferentiation through MITF loss that is mechanistically linked to increased cytokine responsiveness caused by accumulation of c-Jun. This molecular cascade promotes an inflammatory cell state switch that is associated with increased myeloid cell infiltration in human melanoma samples and orchestrates myeloid cell recruitment in syngeneic mouse melanomas. This provides a rational to target myeloid cells in patients with MITF^low^/c-Jun^high^ melanomas.

## Results

### MITF^low^ melanomas have a primed inflammatory cell state

**Figure 1:**
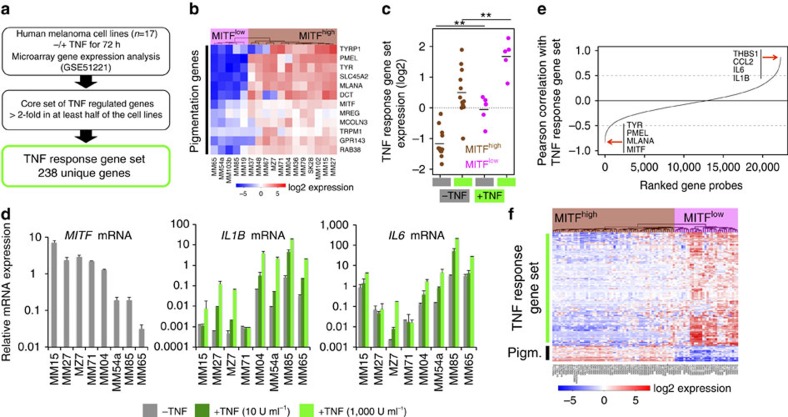
Dedifferentiated melanoma cells have a higher inflammatory responsiveness and pathway activity. (**a**) Outline of bioinformatic analysis. (**b**) Hierarchical clustering and heatmap analysis of melanocytic gene expression to define differentiation status of the melanoma cell lines. Gene expression data were log_2_ transformed and mean centred. (**c**) Expression of the TNF response gene set in TNF-treated (72 h) or -untreated cells grouped by their differentiation status (MITF^high^, differentiated; MITF^low^, dedifferentiated). Significance was determined by analysis of variance and Tukey's honest significant difference test correction for multiple comparisons. **P<0.01. (**d**) Analysis of relative mRNA expression levels on a logarithmic scale by qRT–PCR normalized to ubiquitin (UBC). Cells were treated with different concentrations of TNF for 24 h or left untreated. Error bars indicate s.d. from biological triplicates. (**e**) Correlation of the expression of individual genes with the expression of the TNF response signature in the BROAD melanoma cell line panel (*n*=88). Gene probes were ordered by an increasing Pearson's correlation value. Top negatively or positively correlated genes are highlighted. (**f**) Hierarchical clustering and heatmap analysis of the BROAD melanoma cell line panel using the TNF response gene set and a manually curated pigmentation gene set (abbreviated as Pigm.). Gene expression data were log_2_ transformed and mean centred.

In recent times we analysed the transcriptional response to TNF-α in a panel of well-characterized human melanoma cell lines (*n*=17) (Supplementary Table 1) to identify inflammation-induced migratory gene programmes^4^,^25^,^26^. Here we used this data set to address the interconnection between the melanocytic differentiation status and the inflammatory responsiveness of human melanoma cells (Fig. 1a). We identified a core set of TNF-α responsive genes using cutoff criteria that take the heterogeneity of our panel into account (Methods and Supplementary Data 1). The melanoma cell lines were classified as differentiated (*n*=12) or dedifferentiated (*n*=5) based on the expression levels of *MITF* (MITF^high^ and MITF^low^) and a set of *bona fide* pigmentation genes as measures of the transcriptional activity of MITF using hierarchical clustering (Fig. 1b). We found that TNF-α responsiveness was highly heterogeneous, but MITF^low^ melanoma cell lines had significantly higher inflammatory pathway activity both in the presence and absence of TNF-α (Fig. 1c). These results were validated by quantitative reverse transcriptase–PCR (qRT–PCR) for the TNF-α-inducible genes *IL1B* and *IL6* in independent experiments across a representative subset of melanoma cell lines from our panel (Fig. 1d). Again, higher expression levels of these cytokine genes at baseline correlated with higher expression on TNF-α exposure. This indicates priming of inflammatory pathways and we used this relationship to corroborate our findings in a larger panel of human melanoma cells lines (*n*=88)^27^. We searched for genes that negatively correlated with the expression of the TNF-α response gene signature and we identified MITF and its targets as the top anti-correlated genes (Fig. 1e and Supplementary Data 2). This provides strong evidence for an inverse interrelationship between the melanocytic differentiation status and inflammatory gene programmes in melanoma cells as visualized by a heatmap cluster analysis of the respective cell line panel (Fig. 1f and Supplementary Data 3).

### Acute MITF loss increases global inflammatory responsiveness

**Figure 2:**
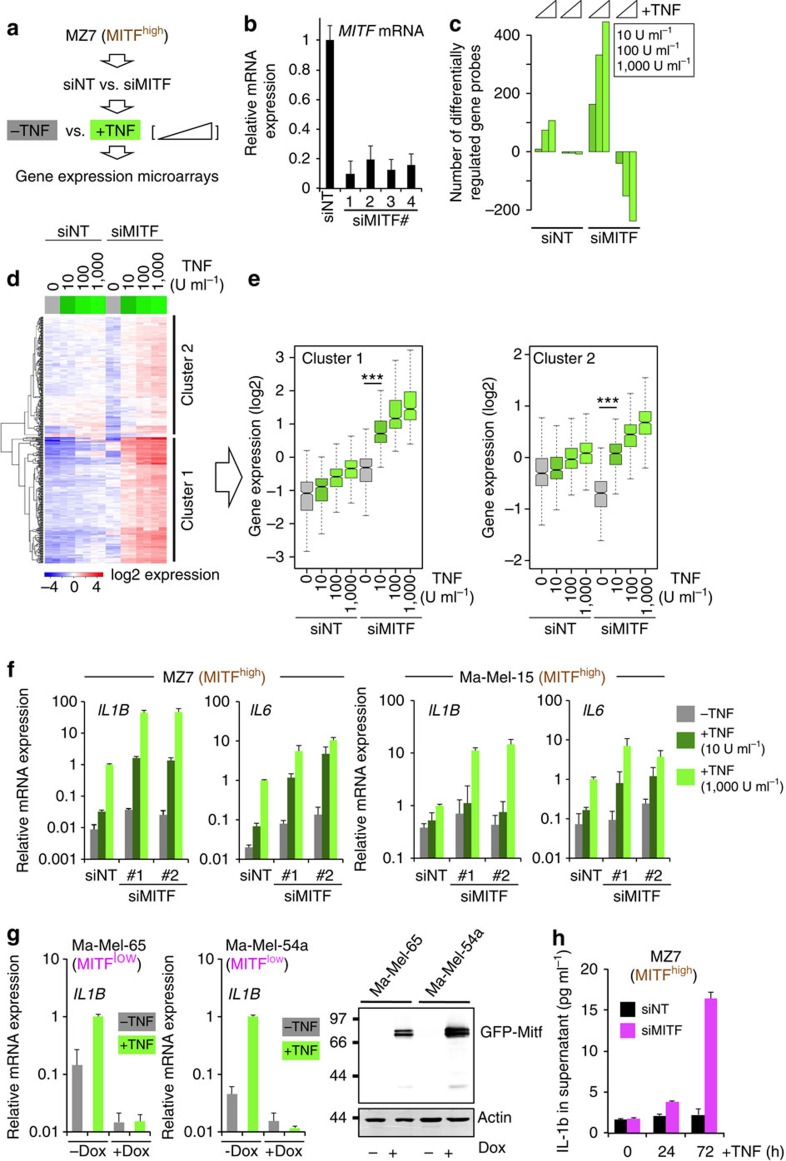
MITF suppresses global inflammatory responsiveness. (**a**) Outline of experimental setup. (**b**) Validation of MITF siRNA knockdown efficiencies by qRT–PCR. siNT represents a pool of non-targeting siRNA controls. Error bars indicate s.d. from technical replicates of a representative experiment (repeated at least three times). (**c**) Quantification of the number of gene probes that were deregulated by at least twofold in response to TNF (24 h) in siMITF- or siNT-transfected MZ7 melanoma cells using gene expression microarrays. (**d**) Gene-probe clustering and heatmap analysis of TNF-induced genes (>twofold). The two main clusters are indicated at the right side. Gene expression data were log_2_ transformed and mean centred. (**e**) Quantification and dissection of the TNF response patterns of the two clusters shown in **d**. Each box represents the distribution of the normalized expression of all genes within the respective cluster. The indicated significance levels were determined by analysis of variance and Tukey's honest significant difference test for correction of multiple comparisons. ***P<0.001. (**f**) Analysis of relative mRNA expression levels by qRT–PCR normalized to ubiquitin (UBC) on a logarithmic scale. Cells were transfected with the indicated siRNAs and treated with different concentrations of TNF for 24 h or left untreated. Error bars indicate s.d. from biological triplicates. (**g**) Left and middle panel: analysis of relative mRNA expression levels on a logarithmic scale by qRT–PCR normalized to UBC in cells with doxycycline (Dox)-inducible MITF expression treated with TNF (24 h). Error bars indicate s.d. from biological triplicates. Right panel: western blotting of Dox-induced GFP–MITF expression detected with anti-MITF. (**h**) ELISA assay for detection of secreted IL-1β from supernatants of TNF-treated MZ7 cells transfected with control (siNT) or siMITF siRNAs. Error bars indicate s.d. from biological triplicates.

As MITF is the key regulator of the melanocytic differentiation pathway, we addressed whether MITF directly influences inflammatory responsiveness by means of RNA interference (RNAi) loss-of-function studies and gene expression microarrays using four equally effective small interfering RNAs (siRNAs) (Fig. 2a,b). Prolonged depletion of MITF can cause a senescence-like response in melanoma cells and it was independently shown that the accumulation of the NF-κB subunit p65/RelA on chromatin of senescent cells is a common phenomenon of cellular senescence also seen in fibroblasts^20^,^28^. Therefore, we performed only short-term depletions of MITF and we verified by immunofluorescent staining that there was no nuclear translocation of p65/RelA by acute MITF loss at this early time point (Supplementary Fig. 1). Next, we determined how MITF suppression changes the global responsiveness of human MZ7 melanoma cells (MITF^high^ group) to increasing amounts of TNF-α. We identified genes with at least twofold change by TNF-α in each of the experimental conditions and found that a reduction of MITF greatly sensitizes to low concentrations of TNF-α and increases the number of TNF-regulated genes (Fig. 2c). Heatmap clustering showed that hyperresponsiveness to TNF-α was not restricted to genes that were either unchanged or moderately induced by MITF loss (cluster 1; Supplementary Data 4), but was also evident for genes (cluster 2; Supplementary Data 5) that were co-suppressed with MITF loss (Fig. 2d). Thereby, TNF counteracted certain parts of the siMITF-mediated transcriptional effects (Fig. 2e, left and right panel). Next, we confirmed these findings by qRT–PCR for the TNF-α responsive genes *IL1B* and *IL6* using independent MITF siRNAs in MZ7 and two other MITF^high^ melanoma cell lines, Ma-Mel-15 (MM15) and Ma-Mel-27 (MM27), respectively (Fig. 2f and Supplementary Fig. 2). In line with our microarray analysis, suppression of MITF consistently caused transcriptional hyperresponsiveness to TNF-α. Inversely, conditional overexpression of MITF (eGFP-MITF^Dox^) in the MITF^low^ cell lines Ma-Mel-65 and Ma-Mel-54a strongly impaired the transcriptional response to TNF-α (Fig. 2g). So far, we have used *IL1B* messenger RNA expression as a marker for inflammatory pathway activation. Interleukin (IL)-1β protein secretion has been reported in some human melanoma cell lines, but many lack detectable levels in the supernatant and consistently we could not detect IL-1β by enzyme-linked immunosorbent assay (ELISA) in TNF-treated or -untreated MZ7 cells^29^,^30^,^31^. However, additional suppression of MITF in the presence of TNF-α resulted in detectable levels of IL-1β in the supernatant in line with an acquired pro-inflammatory cell state (Fig. 2h).

### AP-1 and NF-κB signatures characterize MITF^low^ melanomas

Apart from being a differentiation factor, MITF also functions as a lineage oncogene in melanoma and its acute depletion results in cell cycle arrest^18^,^32^. Gene set enrichment analysis (GSEA) of siMITF-treated MZ7 cells consistently showed pathway alterations associated with cell cycle exit and this was validated by cell growth assays also in other MITF^high^ melanoma cell lines (Supplementary Fig. 3 and Supplementary Data 6 and 7). Nevertheless, the MITF^low^ cell lines from our panel Ma-Mel-85 (MM85), Ma-Mel-54a (MM54a) and Ma-Mel-65 (MM65) proliferated normally in culture even though they expressed MITF at levels far lower than those in siMITF-treated Ma-Mel-15 or MZ7 cells (MITF^high^). Therefore, we asked whether the low residual levels of MITF in the MITF^low^ cell lines were still required for cell cycle progression or not. As knockdown of MITF failed to block their growth we concluded that the residual levels of MITF were dispensable for the proliferation of these MITF^low^ cell lines, suggesting a stable epigenetically distinct and MITF-independent melanoma cell state (Supplementary Fig. 3c).

**Figure 3:**
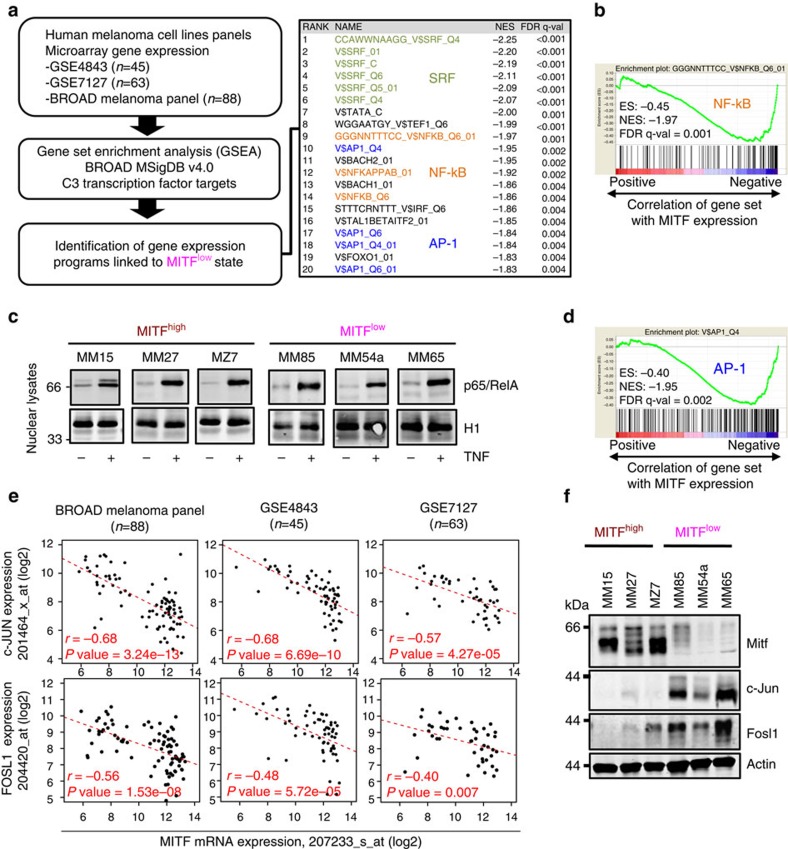
Dedifferentiated melanoma cell state is characterized by reciprocal c-Jun/AP-1 upregulation. (**a**) Left panel: outline of bioinformatic GSEA analysis. Right panel: top 20 enriched gene sets from the GSEA using the MSigDB C3 transcription factor targets gene set collection. (**b**) GSEA plot for the top ranking NF-κB target gene set. ES, enrichment score; NES, normalized enrichment score; FDR, false discovery rate. (**c**) Immunoblot analysis of p65/RelA protein levels in nuclear lysates of indicated cell lines treated with TNF (30 min) or left untreated. The nuclear protein histone H1 was used as loading control. (**d**) GSEA plot for the top ranking AP-1 target gene set. (**e**) Anti-correlation of *JUN* or *FOSL1* mRNA expression with MITF in three independent melanoma cell line panels based on microarray gene expression data (Affymetrix gene probes are indicated). Significance of negative Pearson's correlation values was determined by a two-sided correlation test. (**f**) Immunoblottings for Mitf, c-Jun, Fosl1 and actin in human MITF^high^ and MITF^low^ melanoma cell lines.

As we found that MITF^low^ melanoma cell lines have a higher inflammatory pathway activity and responsiveness, we were interested in the underlying molecular mechanisms. Therefore, we used the transcription factor target gene set collection (C3) of the BROAD Molecular Signature Database (MSigDB v4.0) for GSEA, to identify transcription factor programmes linked to the MITF^low^ cell state in panels of human melanoma cell lines (Fig. 3a). This analysis revealed three predominant and significantly enriched transcription factors SRF, NF-κB and AP-1. The unbiased identification of NF-κB gene sets corroborated an antagonistic relationship between MITF and NF-κB-driven inflammatory pathways (Fig. 3b). Thus, we asked whether MITF^high^ and MITF^low^ melanoma cell lines differ in nuclear levels of the key NF-κB subunit p65/RelA at baseline or in response to TNF-α. Contrary to our expectation, we found that nuclear translocation of p65/RelA was comparable and irrespective of the MITF status (Fig. 3c). Beyond doubt, the transcriptional activity of p65/RelA is fine-tuned by posttranslational modifications, but these results suggested to us that additional mechanisms determine the enhanced inflammatory responsiveness of MITF^low^ melanoma cells.

Therefore, we revisited our transcription factor programme analysis and we were particularly interested in the role of the AP-1 transcription factor complex composed of c-Jun and c-Fos family members, as c-Jun/AP-1 is known to synergize with NF-κB in the transcriptional control of cytokine genes such as *IL1B* (Fig. 3d)^33^. We assorted a list of canonical AP-1 complex family members and calculated their correlation with *MITF* expression levels in three independent panels of human melanoma cell lines (Fig. 3e)^34^. This analysis identified *c-JUN* and *FOSL1* as strongly and consistently anti-correlated genes with *MITF* and we validated the inverse expression pattern of the respective proteins by western blotting in a representative subset of cell lines (Fig. 3f). This suggested that MITF^low^ melanoma cells are characterized by an enforced cooperation between the c-Jun/AP-1 and the NF-κB transcription factor complexes in the control and priming of inflammatory gene programmes.

### MITF suppresses c-Jun through binding to the c-Jun enhancer

**Figure 4:**
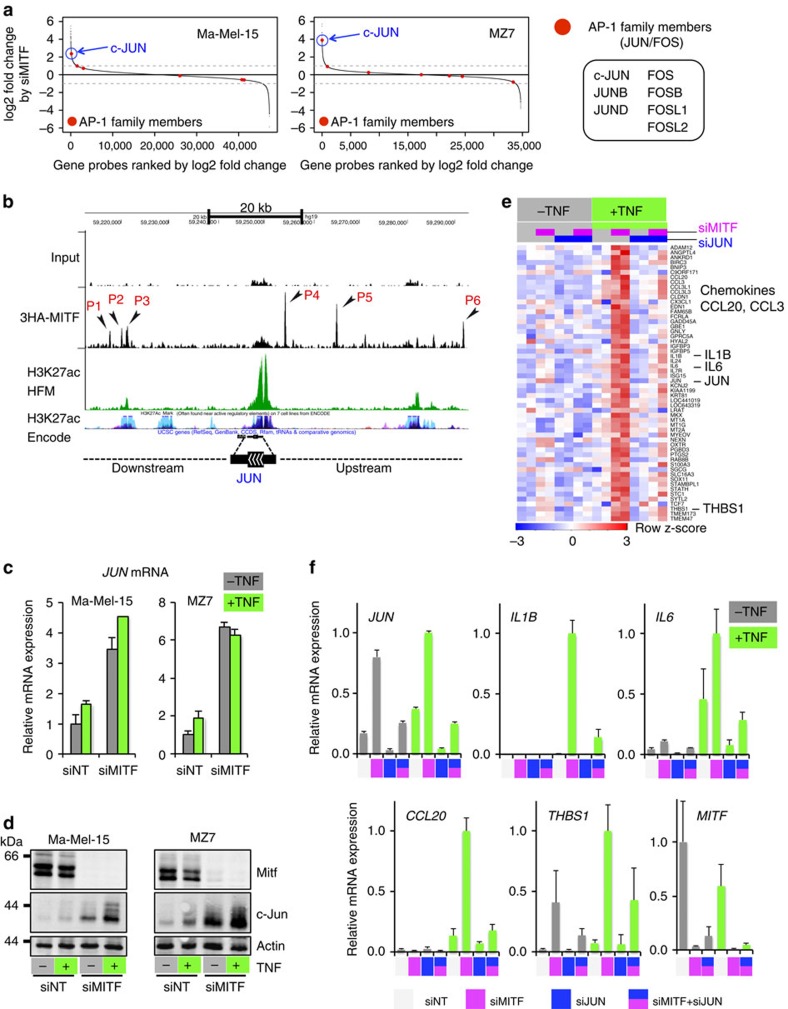
MITF directly suppresses c-Jun that accounts for inflammatory hyperresponsiveness caused by MITF loss. (**a**) Analysis of transcriptional changes of canonical AP-1 family members caused by loss of MITF using microarrays. The quantification is based on two biological replicates for each cell line. (**b**) ChIP-seq profile showing significant 3HA–MITF binding peaks in the *JUN* genomic region (black arrowheads). The input track is shown as a control. Two additional tracks indicate potential enhancer regions (H3K27ac) in human foreskin melanocytes (HFM) and from the Encode project. The scale bar indicates the size of the genomic region in kilobases (kB). (**c**) Analysis of relative *c-Jun* mRNA expression levels by qRT–PCR normalized to ubiquitin (UBC). Cells were treated with TNF for 24 h or left untreated. Values are referenced to siNT/-TNF for each cell line. Error bars indicate s.d. from biological triplicates. (**d**) Immunoblottings for Mitf, c-Jun and actin corresponding to the experiment described in **c**. (**e**) Heatmap analysis of genes strongly dependent on c-Jun in the context of MITF loss and TNF stimulation (24 h) in Ma.Mel15 cells using gene expression microarrays. Candidate genes are highlighted on the right side. (**f**) Independent validation of the heatmap results by qRT–PCR determining relative mRNA expression levels normalized to UBC. Expression values are referenced to the maximum value in each panel. Error bars indicate s.d. from biological duplicates.

Next, we addressed whether there is a direct mechanistic link between MITF and c-Jun/FOSL1 that explains their anti-correlated expression found in melanoma cell line panels. We depleted MITF with siRNA in the two MITF^high^ cell lines MZ7 and Ma-Mel-15 to analyse the transcriptional changes by microarray. We found that *c-JUN* was the only AP-1 family member consistently induced (>twofold) by acute MITF loss (Fig. 4a). This result was independently validated by interrogating our two published RNA sequencing (RNA-seq) data sets (GSE61967) from 501mel melanoma cells that consistently showed upregulation of *c-JUN* on knockdown of MITF (log_2_ fold changes: 1.8/1.4)^19^,^35^. We further asked whether MITF could be directly involved in the transcriptional repression of *c-JUN*. Using our recently described MITF chromatin immunoprecipitation sequencing (ChIP-seq) data set we investigated the MITF binding profile (triple haemagglutinin (HA)-tagged MITF (3HA–MITF)) in the genomic region of *c-JUN* (Fig. 4b)^35^. We identified several significantly enriched MITF-binding sites also containing E-box motifs, a known binding motif of MITF^19^,^35^. Two binding sites (P2 and P3) coincided with an ENCODE-annotated enhancer region (H3K27ac-enriched). ChIP–quantitative PCR experiments in Ma-Mel-65 cells (MITF^low^) conditionally re-expressing MITF (eGFP-MITF^Dox^) largely recapitulated the MITF ChIP-seq profile from 501mel cells, showing prominent binding at P3 in the enhancer region (Supplementary Fig. 4a,b). We devised a CRISPR-Cas9-based genome-editing strategy to target the P3 region right at the E-box motif in its endogenous genomic context (Supplementary Fig. 4a,b)^36^. Ma-Mel-65 (eGFP-MITF^Dox^) cells were transfected with a respective single guide RNA (sgRNA) construct together with mCherry for cell sorting, to enhance genome-editing rates. In an unbiased manner, we isolated 20 single-cell clones and analysed genome editing at the P3 region by next-generation sequencing (NGS) together with suppression of *c-JUN* mRNA on eGFP-MITF induction by doxycycline (Supplementary Figs 4c and 5)^37^. First of all, forced MITF expression reduced *c-JUN* mRNA levels (Supplementary Fig. 4d). Furthermore, and despite an expected heterogeneity of single-cell clone-derived cultures, we found that the majority of cell clones (15/20) with a genome-edited MITF-binding region P3 showed less suppression of *c-JUN* by MITF in comparison with cell clones with a wild-type P3 region (Supplementary Fig. 4d). Of note, one clone with extended deletions of the P3 region on all alleles (NGS results suggested tetraploidy at this locus in Ma-Mel-65 cells) even showed a moderate induction of *c-JUN* by MITF. We verified that all clones potently induced eGFP-MITF on addition of doxycycline to rule out that a lack of MITF induction explains the impaired *c-JUN* repression (Supplementary Fig. 4e,f). Furthermore, eGFP-MITF also reduced c-Jun protein level in a dose-dependent manner (Supplementary Fig. 4g). Altogether, these results further suggest a direct role for MITF in the transcriptional repression of *c-JUN* through binding in the enhancer region, but the precise sequence context required for MITF-mediated inhibition in the *c-JUN* enhancer needs to be clarified by future studies.

### c-Jun suppression by MITF regulates inflammatory responses

Next, we asked how inflammatory TNF-α signalling interconnects with the identified MITF/c-Jun antagonism in melanoma cells. MZ7 and Ma-Mel-15 cells were transfected with siMITF or control siRNAs (siNT, non-targeting) and either stimulated with TNF-α for 24 h or left untreated. Depletion of MITF caused robust accumulation of *c-JUN* mRNA and protein that was further enhanced by TNF-α (Fig. 4c,d). To address the global relevance of c-Jun induction in the context of MITF loss for inflammatory hyperresponsiveness of melanoma cells, we performed co-knockdown experiments with siRNAs against *MITF* and *c-JUN* in the absence or presence of TNF-α and analysed the transcriptional changes by microarray (Fig. 4e). In line with our expectations, the induction of many cytokine or chemokine genes by TNF-α was c-Jun dependent in the context of acute MITF depletion and we independently verified these results by qRT–PCR for a set of selected genes including *IL1B*, *IL6*, *CCL20* and *THBS1*, as well as *c-JUN* and *MITF* as controls for the respective knockdown efficiencies (Fig. 4f).

### c-Jun is critical for inflammation-induced dedifferentiation

**Figure 5:**
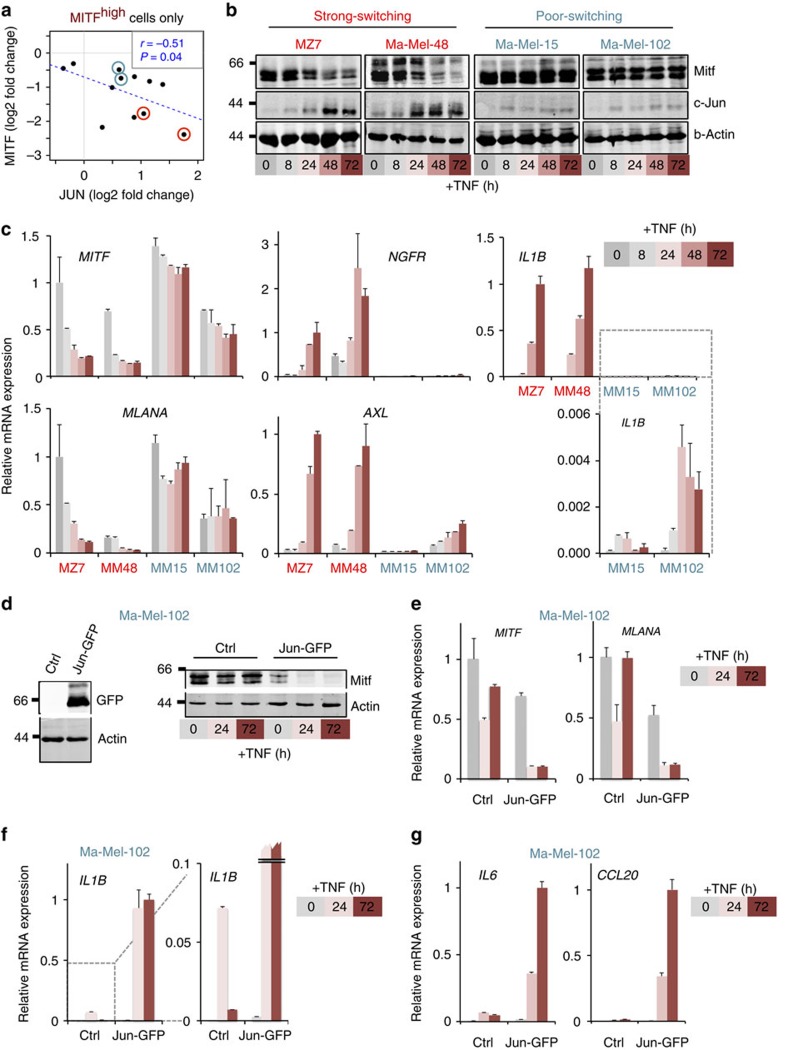
c-Jun is critical for inflammation-induced dedifferentiation and the reciprocal gain of inflammatory responsiveness in melanoma cells. (**a**) Scatter plot of log_2_ fold changes of MITF and c-Jun caused by TNF treatment in MITF^high^ melanoma cell lines based on re-analysis of gene expression data (GSE51221). The significance of negative Pearson's correlation was determined by a one-side correlation test. (**b**) Immunoblottings for Mitf, c-Jun and actin from protein lysates of melanoma cells treated with TNF for the indicated times. (**c**) Experiment as described in **b**, but quantification of relative mRNA expression by qRT–PCR normalized to ubiquitin (UBC) and referenced to the respective highest value of the kinetics in MZ7 cells. Error bars denote s.d. of technical replicates. This representative experiment was performed in parallel to the immunoblot time course shown in **b**. The time courses were independently repeated three times. (**d**) Left panel: immunoblotting of c-Jun-GFP expression in Ma-Mel-102 cells detected by anti-GFP. Right panel: immunoblotting for Mitf and actin in c-Jun-GFP-expressing cells treated with TNF for the indicated times. (**e**–**g**) Experiment as described in **d**, but quantification of relative mRNA expression levels normalized to UBC and referenced to the highest expression value in each panel. Error bars indicate s.d. from biological triplicates.

Next, we dissected the role of the antagonistic MITF/c-Jun interrelationship for the kinetics of inflammation-induced dedifferentiation of melanoma cells. We hypothesized that prolonged TNF-α exposure (72 h) steadily increases *c-JUN* mRNA and protein expression through reduction of MITF and thereby progressively amplifies inflammatory gene programmes in melanoma cells. Re-analysis of the gene expression data from our TNF-stimulated melanoma cell line panel consistently revealed that suppression of *MITF* mRNA significantly correlated with the reciprocal induction of *c-JUN* mRNA, but obviously this switch was variable among the different cell lines (Fig. 5a). For example, MZ7 and Ma-Mel-48 showed profound relative changes in the *MITF*/*c-JUN* mRNA ratio (strong switching), whereas Ma-Mel-15 or Ma-Mel-102 responded moderately (poor switching). We confirmed this heterogeneity by immunoblotting in time-course experiments showing that MITF loss and c-Jun accumulation after TNF-α treatment are tightly coordinated events in melanoma cells (Fig. 5b). Importantly, TNF-α caused p65/RelA translocation and JNK activation in both strong and poor switching cell lines ruling out that major defects in the TNF-α signalling cascade explain the poor switching phenotype (Supplementary Fig. 6a–d). Of note, a recent study suggested that TNF-α could also induce MITF in short-term assays, but none of our 12 differentiated (MITF^high^) melanoma cell lines exhibited MITF upregulation by prolonged TNF-α exposure^38^. As cytokines use common signalling pathways in a redundant manner, we tested several other pro-inflammatory cytokines (interferon-γ, IL-1β, IL-6, IL-8 and chemokine (C-C motif) ligand 2 (CCL2)) and found that in particular IL-1β but also IL-6 reduced MITF expression with reciprocal upregulation of c-Jun (Supplementary Fig. 6e). As IL-1β and IL-6 are *bona fide* TNF-α targets, this delineates a potent feed-forward mechanism of melanoma dedifferentiation instigated by TNF-α.

Subsequently, we analysed the transcriptional changes on TNF-α treatment in strong and poor switching melanoma cell lines in time-course experiments. We investigated selected candidate genes representing markers of both the melanocytic (*MITF* and *MLANA*) and the dedifferentiated cell state (*AXL* and *NGFR*), as well as *IL1B* (Fig. 5c). MZ7 and Ma-Mel-48 (both strong switching) showed a progressive and high induction of *AXL*, *NGFR* and *IL1B* together with an inverse suppression of *MITF* and *MLANA*. In contrast, the poor-switching cell lines Ma-Mel-15 and Ma-Mel-102 exhibited a greatly diminished response to TNF-α with a transient peak of *IL1B* mRNA expression. Peak-like transcriptional responses are typical for the induction of cytokine genes in immune cells to restrain deleterious inflammatory processes through potent counterregulatory mechanisms^39^. Hence, this difference in the kinetics demonstrates how MITF loss overrides intrinsic negative feedbacks of inflammatory signalling pathways and progressively drives the establishment of an inflammatory melanoma cell state.

Given that c-Jun is a canonical component of the TNF-α signalling cascade, we reasoned that the magnitude of c-Jun induction on TNF-α stimulation is crucial for initial MITF reduction to instigate progressive dedifferentiation. As the poor-switching melanoma cell lines Ma-Mel-15 and Ma-Mel-102 hardly induced endogenous c-Jun in response to TNF-α, we stably overexpressed green fluorescent protein (GFP)-tagged c-Jun (Jun–GFP). We found that increased levels of c-Jun reduced the expression of *MITF* and *MLANA*, and importantly this suppression was exacerbated by TNF-α treatment, whereas control cells remained insensitive (Fig. 5d,e). In line with our results, JunB/ATF2 complexes have been previously shown to indirectly inhibit MITF expression through SOX10, a neural crest transcription factor and inducer of MITF^40^. Next, we addressed how enforced c-Jun expression rewired the transcriptional response of TNF-stimulated cytokines by qRT–PCR. We focused on the cytokine genes *IL1B*, *IL6* and *CCL20* that were highly c-Jun dependent in the context of acute MITF depletion by RNAi as shown above (Fig. 4e,f). We found that enforced c-Jun expression turned poor and peak-like inductions of these cytokines into strong, progressive and persistent inductions (Fig. 5f,g). This finding fully establishes the reciprocal regulation between MITF and c-Jun, and it demonstrates that this antagonism determines the intensity, kinetic and duration of the transcriptional response to pro-inflammatory cytokines such as TNF-α in melanoma cells.

### MITF^low^/c-Jun^high^ melanomas are infiltrated by myeloid cells

**Figure 6:**
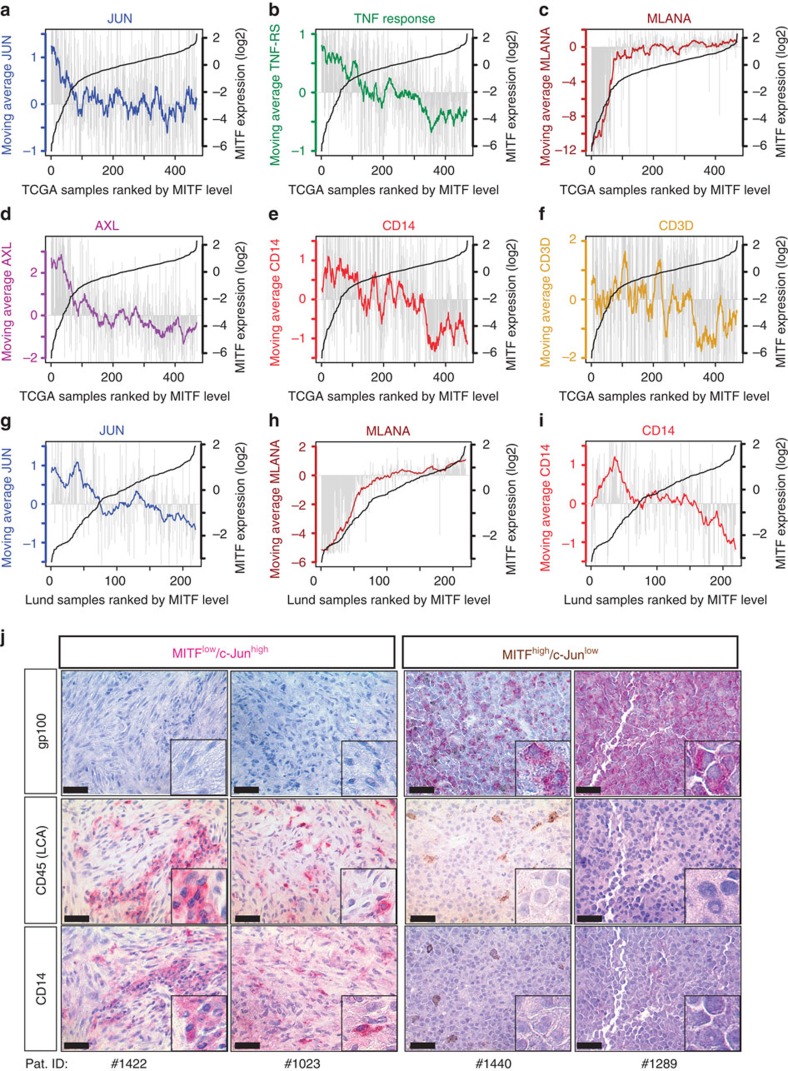
The inflammatory MITF^low^/c-Jun^high^ cell state correlates with increased myeloid cell infiltration in human melanomas. (**a**–**c**) Expression of *c-JUN*, the TNF response gene signature and *MLANA* across TCGA melanoma samples ordered by increasing *MITF* levels from the left to the right in each panel. The black line (corresponding *y* axis at the right side of each panel) shows the increasing MITF expression levels. Grey bars represent the respective expression levels of *c-JUN*, the TNF response signature and *MLANA* in each individual sample. The coloured lines reflect the expression trends as determined by a moving average algorithm with a sample window size of *n*=20. (**d**–**f**) Same analysis as in **a**–**c**, but showing the expression of the melanoma dedifferentiation marker gene *AXL*, the myeloid immune cell marker gene *CD14* and the T-cell gene *CD3D*. (**g**–**i**) Gene expression data of from the Lund melanoma cohort analysed as shown in **a**–**f**. The plots indicate the expression *c-JUN*, *MLANA* and *CD14* in samples ranked by increasing *MITF* level. (**j**) Immunohistochemical analysis for gp100, CD45 and CD14 in representative cases from the Lund melanoma cohort. Small panels show zoom-in views, for example, to visualize morphology of CD45+/CD14+ tumour-infiltrating immune cells. Scale bars, 50 μm.

We consequentially hypothesized that the antagonistic interrelationship between MITF and c-Jun represents an important determinant for the reciprocal cross-talk of melanoma and immune cells in the tumour microenvironment. To address this, we conducted a bioinformatic approach using RNA-seq gene expression data from The Cancer Genome Atlas (TCGA) melanoma cohort (Skin Cutaneous Melanoma, *n*=470) that was accessed through the R-based computing platform of the cBioportal project^41^,^42^,^43^. Melanoma samples were sorted by increasing *MITF* levels and we plotted for each sample the respective expression of *c-JUN*, *FOSL1*, the TNF-α response signature *MLANA* and the dedifferentiation makers *AXL*, *NGFR* and *EGFR* (Fig. 6a–d and Supplementary Fig. 7a–c)^6^,^21^,^24^,^44^. To better visualize interrelationships against the background of heterogeneous human tumour samples, we made use of the large sample size and plotted moving averages of the gene expression levels (Supplementary Software 1). This approach clearly illustrates how the *MITF*^low^/c-*JUN*^high^ switch coincides with the acquisition of a dedifferentiated phenotype in human melanomas as reflected by the loss of melanocytic gene expression (*MLANA*) and the high levels of *AXL*, *NGFR* and *EGFR*. Furthermore, *MITF*^low^/c-*JUN*^high^ melanomas have an increased activity of the TNF response gene set and hence pro-inflammatory pathway activity in line with our results shown so far. However, the TNF response signature contains several cytokine genes that are strongly expressed by immune cells and thus the extent of inflammation and immune cell infiltration contributes to the overall activity of this signature in whole-tumour tissue samples (Supplementary Fig. 7d–f). To explore the immune cell composition in relation to the *MITF*^low^/c-*JUN*^high^ phenotype, we primarily focused on myeloid immune cells and T cells that can be identified by the expression of the marker genes *CD14*, *CSF1R* and *CD3D*, *CD3E*, respectively. We found that *MITF*^low^/c-*JUN*^high^ melanomas have high levels of the myeloid cell transcripts *CSF1R* or *CD14*, arguing that in particular myeloid cell infiltrates are associated with this melanoma phenotype (Fig. 6e,f and Supplementary Fig. 7g,h). Furthermore, gene expression data from a second independent cohort of metastatic melanomas (Lund melanoma cohort, *n*=219) consistently revealed inverse correlations between *MITF* or its target *MLANA* versus *c-JUN* or *AXL* (Fig. 6g,h and Supplementary Fig. 7i)^45^. Consistently, *CD14* and *CSF1R* expression was higher in *MITF*^low^/c-*JUN*^high^ melanomas, indicating increased numbers of myeloid cells (Fig. 6i and Supplementary Fig. 7j). Immunohistochemical analysis of representative cases from this cohort confirmed that *MITF*^low^/c-*JUN*^high^ melanomas were dedifferentiated (gp100^−^) and infiltrated by CD45+ immune cells that were largely CD14+ myeloid cells (Fig. 6j and Supplementary Fig. 7k).

### MITF^low^/c-JUN^high^ mouse melanomas recruit myeloid cells

**Figure 7:**
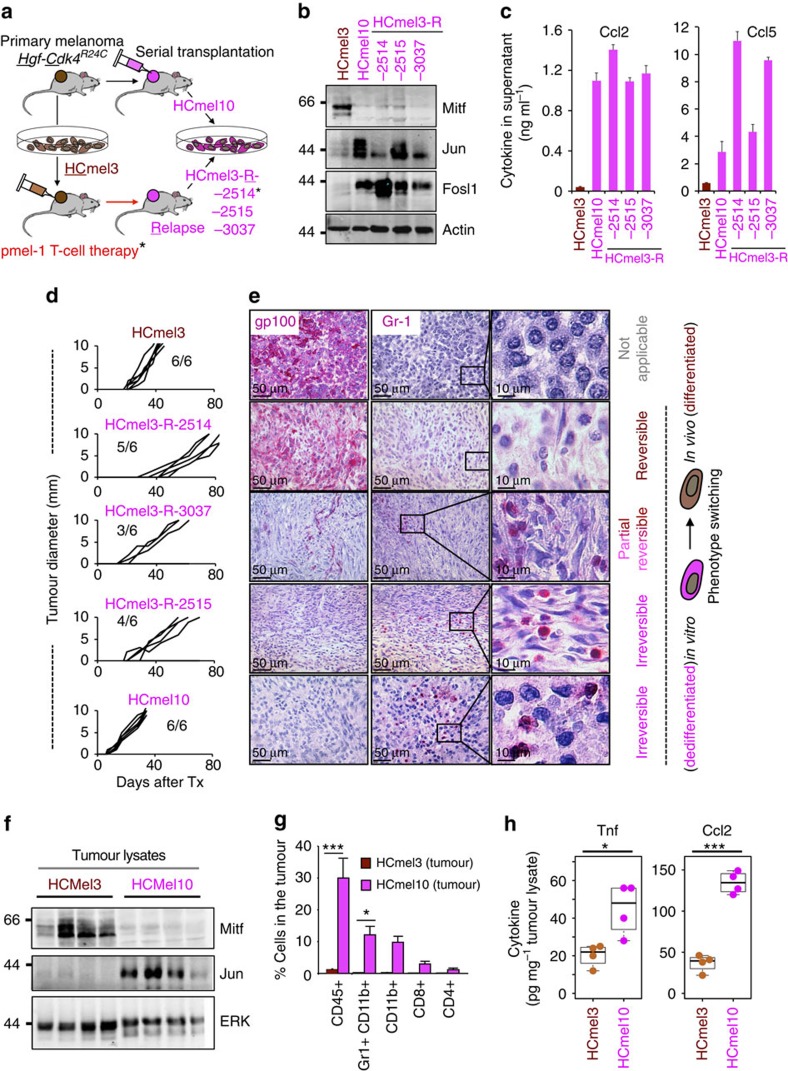
A stable inflammatory MITF^low^/c-Jun^high^ cell state recruits myeloid cells in syngeneic Hgf-Cdk4^R24C^ mouse melanomas. (**a**) Cartoon summarizing the establishment of dedifferentiated mouse melanomas cell lines as described in this and our previous study^6^. (**b**) Immunoblottings showing the expression of Mitf, c-Jun and Fosl1 in the indicated mouse melanoma cell lines. Expression of actin served as loading control. (**c**) Chemokine expression measured by ELISA assay in the supernatant of the indicated mouse melanoma cell lines. Error bars indicate s.d. from biological triplicates. (**d**) Tumour growth kinetics of transplanted HCmel3, HCmel3-R and HCmel10 syngeneic melanomas shown per individual mouse (*n*=6 per group). Tumour growth was measured as diameter. Numbers (for example, 3/6) indicate tumour take rate (3 out of 6). (**e**) Immunohistochemical analysis for gp100 (melanocytic differentiation marker) and Gr-1 (myeloid cell marker) in the respective syngeneic melanomas shown in **d**. Size bars indicate the magnification of the pictures in each panel (50 and 10 μm). The panels at the right show a zoom-in view of the Gr-1 stain, to visualize morphology of individual Gr-1+ myeloid immune cells. (**f**) Immunoblottings for Mitf, c-Jun and actin from HCmel3 and HCmel10 tumour lysates. (**g**) Summary of flow cytometry analysis to quantitatively characterize tumour-infiltrating immune cells in Hcmel3 and HCmel10 syngeneic melanomas (*n*=3 in each group, mean percentage±s.e.m., unpaired two-tailed Student's *t*-test; *P<0.05, ***P<0.001). (**h**) ELISA assay for Tnf and Ccl2 from tumour lysates from HCmel3 and HCmel10 melanomas. (*n*=4 in each group, unpaired two-tailed Student's *t*-test; *P<0.05, ***P<0.001; horizontal lines and whiskers indicate quartiles).

Finally, we aimed to address whether the MITF^low^/c-JUN^high^ melanoma cell state actively orchestrates myeloid cell recruitment *in vivo*. In our previous study we isolated a series of dedifferentiated mouse melanoma cell lines (designated as HCmel3-R) from HCmel3 syngeneic melanomas that had escaped from one or even two cycles of adoptive T-cell therapy directed against the melanocytic antigen gp100 (Fig. 7a and Supplementary Table 2)^6^. The parental differentiated HCmel3 cell line (gp100^+^) was established from a primary melanoma arising in the *Hgf-Cdk4*^R24C^ genetically engineered mouse model and syngeneic HCmel3 melanomas closely resemble pigmented human immune-cell-poor melanomas^6^,^46^. In that study, we reported the unexpected finding that some of the dedifferentiated (*in vitro*) HCmel3-R cell lines (gp100^−^) re-differentiated and re-expressed gp100 on transplantation into new recipient mice restoring responsiveness to the gp100-directed T-cell therapy. Here we characterize the full spectrum of phenotypic heterogeneity observed in an extended panel of HCmel3-R cell lines (HCmel3-R-2514, −2515 and −3037) and the syngeneic melanomas derived thereof. In addition, we have independently established a spontaneously dedifferentiated mouse melanoma cell line (designated as HCmel10) by serial transplantation of a primary pigmented *Hgf-Cdk4*^R24C^ melanoma (Fig. 7a and Supplementary Fig. 8a).

All dedifferentiated mouse melanoma cell lines (HCmel3-Rs and HCmel10) expressed low amounts of MITF and its targets, but high levels of c-Jun/Fosl1, showing that the MITF^low^/c-Jun^high^ cell state is conserved in mouse melanomas (Fig. 7b and Supplementary Fig. 8b). As we hypothesized that the MITF^low^/c-Jun^high^ cell state promotes myeloid cell recruitment, we analysed the expression of major myeloid cell chemoattractants (Ccl5, Ccl2 and Cxcl10) by ELISA assay and qRT–PCR analysis. Indeed, the dedifferentiated HCmel3-R and HCmel10 cell lines expressed and secreted high amounts of these chemokines in comparison with HCmel3 (Fig. 7c and Supplementary Fig. 8c,d). Of note, conditional re-expression of MITF in HCmel3-R cell lines suppressed c-Jun and chemokine expression, corroborating that MITF directly counteracts the inflammatory cell state in murine melanomas as well (Supplementary Fig. 8e,f). Next, we injected HCmel3, HCmel3-Rs and HCmel10 cell lines into the flank of syngeneic mice and monitored tumour growth (Fig. 7d). Some HCmel3-R cell lines showed a slightly reduced tumour take and growth rate. Immunohistochemical analysis for the melanocytic antigen gp100 and the myeloid cell marker Gr-1 revealed that syngeneic HCmel3-R-2514 melanomas re-differentiated and re-expressed gp100, but lacked immune cell infiltrates (Fig. 7e). In contrast, HCmel3-R-3037, HCmel3-R-2515 and HCmel10 melanomas remained largely negative for gp100. Gr-1+ myeloid cells were frequently found within these tumours, demonstrating that a stable dedifferentiated melanoma cell state promotes myeloid cell recruitment *in vivo*, whereas redifferentiation results in an immune-cell-poor melanoma (HCmel3-R-2514). Quantitative FACS-based analysis confirmed the myeloid-cell-rich phenotype (Gr-1+/CD11b+ or CD11b+) of stably dedifferentiated syngeneic melanomas that also showed higher levels of Tnf and its downstream target Ccl2 as shown by ELISA assay of tissue lysates (Fig. 7f–h and Supplementary Fig. 9a). Altogether, these findings support our model that an inflammatory melanoma cell state controlled by the antagonistic interrelationship between MITF and c-Jun fosters reciprocal interactions with myeloid immune cells (Supplementary Fig. 9b).

## Discussion

Phenotypic heterogeneity of tumour cells arises from genetic and epigenetic changes^47^,^48^. Even cultured cancer cell lines show phenotypically distinct subpopulations that dynamically interconvert and their relative frequencies rapidly respond to perturbations by cancer drugs or inflammatory signals^6^,^49^,^50^. This tumour cell intrinsic plasticity is tightly interconnected with the microenvironment, and hence inflammation or altered metabolic states such as hypoxia profoundly shape the phenotypic landscape of cancers^7^,^13^. Heterogeneity of the immune cell composition in tumours emerges as an important determinant for cancer immunotherapies that have recently achieved significant clinical breakthroughs^51^,^52^,^53^. Further, immune cell infiltrates are subject to dynamic changes during treatment in both melanoma patients and preclinical mouse models^46^,^52^. As the communication between melanoma and immune cells is reciprocal, tumour phenotypes shape the immune landscape and vice versa^4^,^6^. On the other hand, tumour cells may also require barriers to restrain the impact of plasticity inducing microenvironmental signals, to sustain certain cell states that optimally support proliferation and survival.

Our present study identified a reciprocal and antagonistic interrelationship between MITF and c-Jun as a molecular interface that orchestrates melanoma phenotype switching with inflammatory signals from the microenvironment. The NF-κB and c-Jun/AP-1 transcription factor complexes are key components of the TNF-α signalling cascade, a major pro-inflammatory cytokine, and they synergize in the induction of cytokine genes. Our findings suggest that MITF directly suppresses c-Jun through binding in its regulatory enhancer region and thereby negatively controls priming of inflammatory pathways in melanoma cells. Furthermore, c-Jun engagement by TNF-α and other cytokines such as IL-1β and IL-6 is critical to trigger inflammation-induced dedifferentiation of melanoma cells and the progressive acquisition of a pro-inflammatory cell state that characterizes MITF^low^/c-Jun^high^ melanoma cell lines and tumours. The reciprocity of c-Jun induction and MITF suppression determines the kinetics of the expression of TNF-α-induced cytokine genes and turns a poor and peak-like transcriptional response into a strong, progressive and persistent response. Hence, our work shows that the melanocyte lineage transcription factor MITF acts as a barrier to pro-inflammatory cytokines through inhibition of c-Jun, a general mediator of stress signals. This may protect the MITF-driven proliferative cell state in melanoma cells against transient inflammatory changes in the tumour microenvironment. Physiologically, this mechanism may also help normal melanocytes to enforce pigment production in the inflamed sunburned skin to ensure adaptive tanning, as c-Jun is strongly activated by ultraviolet irradiation^54^,^55^. On the contrary, rewired ERK/JNK signalling in melanoma cells provides an explanation for the increased susceptibility to plasticity-inducing inflammatory cytokines and the JunB-ATF2 axis may also prime melanoma cell plasticity through suppression of Sox10/MITF^40^,^56^. Posttranslational stabilization of c-Jun by E-cadherin loss during disease progression may represent a synergistic route to increased cell state switching in melanoma^57^. In line with our results, a recent epigenetic study identified TEAD and AP-1 transcription factors as drivers of the ‘invasive' and BRAF inhibitor-resistant melanoma cell state^58^. Thus, our work provides a molecular framework that links inflammation-induced dedifferentiation and the transition towards a stable ‘invasive' melanoma cell state.

Inverse correlations between MITF- and NF-κB activity have been described in other contexts such as the resistance to BRAF inhibitors or senescence-like cell states provoked by prolonged depletion of MITF in MITF^high^ melanoma cell lines^19^,^20^,^23^,^59^. However, the molecular mechanisms remain unclear how MITF^low^ melanoma cell lines become independent from the MITF-driven proliferative programme, assuming that they underwent a phenotypic transition and originated from a MITF^high^ cell state. Several transcription factors such as Gli2, Tcf4 and Brn-2 have been implicated in the control of MITF-opposing gene programmes, but it is not known whether their expression is sufficient to immediately compensate for an acute and complete loss of MITF in MITF^high^ melanoma cell lines^10^,^11^,^60^. We believe that regenerative inflammatory microenvironments and hypoxia represent two major conditions that favour stable transitions of melanoma cell states on prolonged exposure through widespread epigenetic remodelling^6^,^7^,^13^,^14^,^16^. The recently described co-occurrences of hypermethylated promoters of both *MITF* and its melanocytic target genes such as *MLANA* support a model that requires a prolonged period of global cell state reprogramming to consolidate this phenotype switch in human melanomas^61^.

Recent studies including ours have demonstrated that mouse models help to elucidate species-conserved mechanisms of melanoma cell plasticity^6^,^62^,^63^. Previously, we showed that an adoptive transfer of cytotoxic T cells creates a chronic inflammatory and immune-cell-rich microenvironment that drives melanoma dedifferentiation^6^. The association of an amelanotic phenotype with an inflammatory myeloid-cell-rich microenvironment has also been found in other genetically engineered mouse melanoma models, supporting the concept of a common underlying mechanism^62^,^63^. A recent study suggested that tumour cell-intrinsic β-catenin signalling is a critical determinant of T-cell exclusion in melanoma^64^. In the present study we demonstrate that dedifferentiated mouse melanoma cells recapitulate the MITF^low^/c-Jun^high^ phenotype-like subsets of human melanomas with increased inflammatory responsiveness and chemokine expression. Hence, the antagonistic relationship between MITF and c-Jun represents a species-conserved molecular framework governing melanoma cell plasticity in response to inflammation. We also show that a stable dedifferentiated and inflammatory melanoma cell state promotes the recruitment of myeloid immune cells into the tumour microenvironment of syngeneic melanomas, whereas *in vivo* redifferentiation results in immune-cell-poor melanomas. Future studies are required to identify the presumably epigenetic determinants that either drive or prevent the reversibility of a dedifferentiated melanoma cell state on re-transplantation of the cells *in vivo*. In line with our mouse models, gene expression and immunohistochemical analysis of human melanoma cohorts revealed that the MITF^low^/c-Jun^high^ molecular switch coincided with an increase of both myeloid immune cells and inflammatory pathway activity.

As tumour-infiltrating myeloid cells such as macrophages support tumour growth and exert immunosuppressive functions, they represent attractive targets for pharmacological intervention in combination with current immunotherapeutic strategies such as immune checkpoint inhibitors^30^,^65^,^66^,^67^,^68^. Our work identified melanoma cell dedifferentiation as a molecular feed-forward mechanism that enforces reciprocal interactions of melanoma and myeloid immune cells. This provides a rational to develop strategies targeting the MITF^low^/c-Jun^high^ melanoma cell state in combination with myeloid cell-directed therapies.

## Methods

### Cell culture of melanoma cell lines

All melanoma cell lines were maintained in RPMI 1640 containing 100 U ml^−1^ penicillin and 100 μg ml^−1^ streptomycin, 2 mM L-glutamine and 10% fetal bovine serum. HEK 293T cells were used for retrovirus and lentivirus production, and were maintained in DMEM medium containing the same supplements (all media and supplements by Life Technologies). All cells were grown in a humidified incubator with 5% CO_2_ at 37 °C. All cell lines were negative for mycoplasma and routinely tested on a monthly basis. None of the cell lines used in this study is listed in the ICLAC list of misidentified cancer cell lines. All Ma-Mel human melanoma cell lines were kindly provided by Dirk Schadendorf (University Hospital Essen, UKE, Germany). The Ma-Mel cell lines were established and characterized by the laboratory of Dirk Schadendorf. MZ7 cells were obtained from Thomas Wölfel (Mainz, Germany). Key driver mutations were confirmed by Sanger sequencing and BRAF mutations were functionally addressed by sensitivity to BRAF inhibitors. Gene expression profiles of untreated cultures were compared with a previous study (GSE4843) and showed consistent results.

For siRNA tranfections, 300,000 cells were seeded per well in a 12-well plate and reverse transfected with 30 pmol siRNA (Dharmacon) using 3 μl RNAiMAX Lipofectamine (Life Technologies) according to the manufacturer's instructions. The medium was changed after 24 h. All recombinant human cytokines were from Peprotech and were used at the following concentrations: CCL2 100 ng ml^−1^, interferon-γ 1,000 U ml^−1^, IL-1β 1,000 U ml^−1^, IL-6 1,000 U ml^−1^, IL-8 150 ng ml^−1^, TNF-α 1,000 U ml^−1^, if not indicated otherwise. For colony formation assays, cells were fixed with 4% formaldehyde and stained for 20 min with 0.05% crystal violet. Plates were scanned using the Odyssey Sa Imaging System (LI-COR Biosciences) at 800 nm.

### Generation of viral transduction particles

For the generation of retroviral transduction particles, HEK 293T cells were transfected with the packaging constructs (gag-pol and pCMV VSV-G) and the transfer plasmid by calcium phosphate transfection. For lentiviral particles, HEK 293T cells were transfected with third-generation lentiviral packaging (pMDLg/pRRE, pRSV-Rev and pMD2.G) and the lentiviral expression plasmid. The medium was refreshed after 24 h. The following day, supernatants were filtrated using a 0.45 μM syringe filter and added to the target cells. After 48 h, the cells were subjected to antibiotic selection using 2 μg ml^−1^ puromycin or 10 μg ml^−1^ blasticidin (both Sigma-Aldrich) for ∼5 days. The complementary DNA encoding c-Jun was amplified from a LentiORF cDNA construct (Dharmacon) and cloned into the retroviral plasmid pRp (kindly provided by Eicke Latz, Institute of Innate Immunity, Bonn, Germany) using BamHI and NheI/AvrII restriction sites. MITF-M cDNAs were kindly provided by Shigeki Shibahara (pRC/CMV MITF-M; Tohoku University School of Medicine, Sendai, Japan) and David Fisher (pLNCX2-MITF-M; Massachusetts Generel Hospital, Boston, USA). A variant of the tet-inducible lentivirus pLV-tetO and the transactivator rtTA (FUdeltaGW-rtTA) were kindly provided by Jochen Uttikal (Deutsches Krebsforschungszentrum, Heidelberg, Germany). For the generation of an inducible lentiviral GFP-MITF-M construct, MITF-M cDNA was first cloned into pEGFP-C2 (Clontech) using HindIII and SalI restriction sites, to generate a fusion cDNA. GFP-MITF-M was amplified from this construct and cloned into pLV-tetO via SalI and MluI sites. rtTA cDNA was amplified from FUΔGW rtTA and cloned into pRp using HindIII. For the generation of cell lines expressing tetracyclin-inducible GFP-MITF, cells were first infected with pRp rtTA and selected with puromycin. Second, cells were transduced with the tet-inducible lentiviral constructs and selected with blasticidin. Gene expression was induced with 0.5 μg ml^−1^ doxycycline (Sigma-Aldrich).

### RNA isolation and quantitative real-time PCR

Cells (0.2–5 × 10^6^) were lysed in RLT buffer (Qiagen) and total RNA was extracted using Zymo Spin II columns (Zymo Research) and wash buffers RW1 (Qiagen), and Zymo RNA Wash Buffer (Zymo Research). The cDNA synthesis reaction was performed in a volume of 10 μl using 0.5–1 μg RNA and 100 U RevertAid Transcriptase, 100 ng Random Hexamer Primer and 10 U RiboLock Rnase Inhibitor (all reagents from Thermo Scientific). The reaction was incubated at 42 °C for 60 min followed by an inactivation step at 72 °C for 10 min. All quantitative real-time PCR reactions were carried out in technical duplicates (in addition to the biological replicates) in a volume of 10 μl using EvaGreen (BioBudget) on the LightCycler 480 System (Roche) according to the manufacturer's instructions. Samples were quantified by normalization to the standard housekeeping gene Ubiquitin. Sequences of all primer pairs used in this study are listed in Supplementary Table 3.

### Chromatin immunoprecipitation

For the ChIP assay, Ma-Mel-65 with inducible Mitf-GFP expression and control cells were grown on five 15-cm dishes each, treated with doxycyclin for 24 h and harvested at 90% confluency. Chromatin was prepared using the SimpleChIP Enzymatic Chromatin IP Kit (Cell Signaling) according to the manufacturer's instructions. A Branson Analog Sonifier 250 was used to disrupt the nuclear membranes, using a microtip and three sets of 20-s pulses with 20% duty cycle and output control set on 2. Chromatin containing 15 μg DNA was immunoprecipitated using 30 μl GFP-trap MA beads (Chromotek) in a total volume of 500 μl. The purified DNA was analysed by quantitative PCR. Sequences of all primer pairs used in this study are listed in Supplementary Table 3.

### Generation of sgRNA CRISPR-Cas9 plasmid

px330-U6-Chimeric_BB-CBh-hSpCas9 (px330) (Addgene plasmid #42230) was digested with BbsI and gel purified^36^. A DNA oligonucleotide (Microsynth) was cloned into the digested pX330 vector, to generate an sgRNA against the MITF-ChiP-seq peak 3 (E-box) in the enhancer region of the human *c-JUN* locus (target sequence with E-box and protospacer adjacent motif (PAM)). Design rules were used as described at http://www.genome-engineering.org/). Sequences of DNA oligos are listed in Supplementary Table 3.

### Next-generation sequencing

Genomic DNA from cultured Ma-Mel-65 cell clones was extracted using the Nucleo Spin Tissue kit (Macherey&Nagel) according to the manufacturer's recommendations. For the generation of targeted PCR amplicons for NGS, a two-step PCR protocol was performed. For the first PCR, gene-specific primers (encompassing the sgRNA target site) were used with additional adapter sequences at both sides of the resulting amplicon. Sequences of all primer pairs used in this study are listed in Supplementary Table 3. Adapter-specific primers containing a barcode sequence and the Illumina adapter sequences P5 and P7 were used for the second PCR (combinations of D5XX and D7XX primers; Supplementary Table 3). In the first PCR, the genomic region of interest was amplified with 18 cycles, using ∼20–50 ng of genomic DNA as input and Phusion HD Polymerase (New England Biolabs) in a 12.5-μl mixture according to the manufacturer's protocol. Next, 2 μl were transferred to the second PCR. This product was amplified with another 18 cycles in a 25-μl reaction mix with Phusion HD Polymerase. NGS sequencing was performed with MiSeq Gene and Small Genome Sequencer (Illumina) according to manufacturer's standard protocols with a single-end read and 300 cycles (MiSeq Reagent Kit v2 300 cycle). Indel analysis was performed using the web-based Outknocker programme (http://www.outknocker.org/).

### Immunoblot analysis

Total cell lysates were prepared by lysing cells in 1 × Laemmli buffer (1,000 cells per μl) and incubation for 3 min at 95 °C. For the separation of nuclear and cytoplasmic fractions, the NE-PER kit (Thermo Scientific) was used according to the manufacturer's instructions, with one additional PBS wash of the nuclear pellet. The lysates were separated using 10% SDS–PAGE and transferred to a nitrocellulose membrane (GE Healthcare) by wet blotting for 80 min at 70 V (both systems by BioRad). After blocking for 1 h with 5% BSA (GE Healthcare) in TBS with 0.5% Tween, membranes were probed with primary antibodies overnight at 4 °C in 2.5% BSA in TBS with 0.5% Tween. The proteins were detected using IRDye680LT and IRDye800CW secondary antibodies and the Odyssey Sa Imaging system (LI-COR Biosciences). The following antibodies were used: β-actin (clone C4, Santa Cruz sc-47778, 1:1,000), histone H1 (clone AE-4, Santa Cruz sc-8030, 1:250), Mitf (Atlas Antibodies, HPA003259, Sigma-Aldrich, 1:250), c-Jun (clone 60A8, #9165 Cell Signaling, 1:250), p65/RelA (D14E12, Cell Signaling #8242, 1:1,000), Fosl1/Fra1 (clone R-20, Santa Cruz sc-604, 1:250), β-tubulin (clone D2N5G, Cell Signaling #15115, 1:2,000), IκBa (clone L35A5, Cell Signaling #4814, 1:1,000), JNK (polyclonal, Santa Cruz sc-571, 1:250), phosphoJNK (polyclonal, R&D AF1205, 1:250), ERK (p42/44 mitogen-activated protein kinase, polyclonal rabbit, #9102, Cell signaling 1:1,000); ladder: Broad Range Marker (Santa Cruz sc-2361). Full blottings corresponding to the immunoblottings shown in the main figures are provided as Supplementary Figs 10–13.

### Immunofluorescence staining

Cells were seeded on coverslips and treated as indicated. Fixation was done with 4% p-formaldehyde (Carl Roth) for 10 min at room temperature, followed by three short washes with PBS and 10 min permeabilization with 0.04% Triton X-100 in PBS (PBS/Triton). Fixed cells were blocked with 10% FCS in PBS for 45 min at room temperature and probed with the primary antibodies (1:100) overnight at 4 °C in a wet chamber. After three washes with PBS/Triton, the coverslips were incubated with the secondary antibody (1:300) for 45 min at room temperature. The coverslips were washed twice with PBS/Triton and incubated with 4,6-diamidino-2-phenylindole in PBS (0.5 μg ml^−1^) for 1 min, followed by one final wash with PBS. Cells were mounted on object slides using Fluoroshield mounting medium (Sigma-Aldrich) and analysed on a Fluorescence microscope using an oil-immersion objective. Primary antibodies were as follows: MITF (C5, sc-56725 Santa Cruz) and p65/RelA (D14E12, #8242 Cell Signaling). Secondary antibodies were as follows: donkey anti-mouse IgG-fluorescein isothiocyanate and donkey anti-rabbit IgG-Texas Red (sc-2099 and sc-2784, Santa Cruz).

### ELISA assay

Supernatants of stimulated cells grown to 90% confluency were collected and centrifuged at 300 *g* for 5 min. Secreted IL-1β was assayed using the BD OptEIA Human IL-1β ELISA Set II (BD Biosciences). The following ELISA assays were used for mouse cytokines in melanoma cell supernatants: CCL5 (R&D DuoSet catalogue number DY478), CCL2 (R&D DuoSet catalogue number DY479) and CXCL10 (R&D DuoSet catalogue number DY466). The absorbance was measured using the Epoch Microplate Spectrophotometer (BioTek). For wavelength correction, readings at 570 nm were subtracted from the readings at 450 nm. Mouse melanoma samples were collected and immediately snap frozen in liquid nitrogen. The levels of TNF in melanoma tissue lysates were measured using the following ELISA (R&D, Mouse TNF-alpha Quantikine #MTA00B). Frozen tumour tissue was ground using a mortar and pestle, and lysed in M-PER Mammalian Protein Extraction Reagent (Life Technologies). Lysates were cleared according to the manufacturer's instructions. The protein concentration was determined using a Bradford assay (Roti-Quant, Carl Roth) and 250 μg total protein were used for each well.

### Tumour transplantation experiments

The HCmel3 cell line was generated from a primary DMBA-induced Hgf-Cdk4^R24C^ melanoma. The HCmel10 cell line was generated by serial transplantation from a spontaneous cutaneous Hgf-Cdk4^R24C^ melanoma. Briefly, tumours were harvested, dissociated mechanically, incubated with 1 mg ml^−1^ of collagenase D (Roche) for 30 min at 37 °C and filtered through 70-μm cell strainers (BD Biosciences). Cells (10^6^) were seeded into collagen-coated six-well plates and cultured in complete RPMI 1640 medium supplemented with 10% FCS (Biochrome), 2 mM L-glutamine (Gibco), 10 mM non-essential amino acids (Gibco), 1 mM HEPES (Gibco), 20 μM 2-mercaptoethanol, 100 IU ml^−1^ penicillin and 100 μg ml^−1^ streptomycin (Invitrogen). HCmel3-R cell lines (HCmel3-R-3037, HCmel3-R-2514 and Hcmel3-R-251515) were established similarly from pmel-1 T-cell therapy relapsed HCmel3 melanomas. Groups of syngeneic C57BL/6 (H-2b) 8- to 10-week-old female mice (purchased from Charles River) were injected intracutaneously with 4 × 10^5^ HCmel3, HCmel3-R or HCmel10 melanoma cells into the flanks. The tumour size was measured twice weekly and recorded as mean diameter in millimetres. Mice with tumours >10 mm were killed. Experiments were performed in groups of six mice. On the basis of previous and additional preparatory experiments concerning FACS-based analysis of immune cell contents in mouse melanomas, we used the observed mean numbers and variances to estimate sample sizes using the R function power.*t*.test (parametric two-sided *t*-tests with *α*-error probability=0.05, power=0.95). All animal experiments were conducted on the C57BL/6 background according to the institutional and national guidelines for the care and use of laboratory animals with approval by the local government authorities (LANUV, NRW, Germany).

### Histology and immunohistology

Human melanoma tissues were obtained from the Department of Oncology at Lund University. Informed consent was obtained and the study was previously approved by the Regional Ethics Committee at Lund University (Dnr. 191/2007 and 101/2013). Mouse tissue samples were immersed in a zinc-based fixative (BD Pharmingen) and human melanoma samples in buffered paraformaldehyde (Dako). Tissues were embedded in paraffin and sections stained with H&E according to standard protocols. Immunohistochemistry was performed with rabbit anti-mouse gp100 pAb (NBP1–69571; Novus Biologicals, dilution 1:400), rat anti-mouse Gr-1 mAB (Clone RB6–8C5, BD Pharmingen/Biosciences, dilution 1:500), mouse anti-human HMB45 mAB (ENZ-C34930, Enzo Life Sciences, dilution: 1:200), mouse anti-human CD45/LCA mAB (Clone 2B11, DAKO), rabbit anti-human CD14 mAB (clone EPR3652, LifeSpan Bio Sciences, dilution: 1:25), mouse anti-human MITF mAB (Clone C5, Thermo Scientific, dilution: 1:50) followed by enzyme-conjugated secondary antibodies and the LSAB-2 colour development system (Dako). Heavily pigmented mouse melanomas were bleached before staining (20 min at 37 °C in 30% H_2_O_2_ and 0.5% KOH, 20 s in 1% acetic acid and 5 min in TRIS buffer). Stained sections were examined with a Leica DMLB immunofluorescence microscope. Images were acquired with a JVC digital camera KY-75FU and processed with Adobe Photoshop.

### Flow cytometry (FACS) of tumour tissues

Transplanted melanoma infiltrating immune cells were isolated as described previously and stained with fluorochrome-conjugated monoclonal antibody specific for mouse CD45 (clone 30-F11, 1:200), CD11b (clone M1/70, 1:200), Gr1 (clone RB6–8C5, 1:200), CD8a (clone 53-6.7, 1:200) and CD4 (clone GK1.5, 1:200; all from BD Pharmingen) according to standard protocols. Data were acquired with a FACSCanto Flow Cytometer (BD Biosciences) and analysed with FlowJo software (TreeStar, V7.6.5 for Windows).

### Microarray analysis of gene expression

RNA from melanoma cell lines was isolated as described above, quantified fluorimetrically and assayed for integrity with the Agilent Bioanalyser (Agilent Technologies). One hundred nanograms of RNA was converted to biotinylated cRNA using one round of amplification with the Illumina Labelling Kit (Illumina) and one round of T7 polymerase amplification and hybridized to Illumina Human Beadchips HT12 v4.0. After hybridization and staining, the arrays were scanned in an Illumina Bead Station and the images processed using Illumina Bead Studio software. Raw microarray data were extracted from the Illumina BeadStudio software and imported into the R statistical programming environment and the Bioconductor platform using the beadarray package. Variance stabilization (vsn package) and normalization including log_2_ transformation of gene expression values were performed, followed by quality assessment of the fit. Gene expression data have been deposited in the GEO database with the following accession numbers: GSE71798, GSE71881 and GSE71886. Central R-based computer code is provided as Supplementary Software.

### Gene expression analysis of melanoma cell line panels

Affymetrix raw CEL files from the BROAD melanoma cell line panel of 88 melanoma cell lines were downloaded from the BROAD melanoma portal (https://www.broadinstitute.org/software/cprg/?q=node/46) and normalized by Robust Multichip Average using the affy package. The log_2_-transformed gene expression values were mean centred for heatmap visualization. Gene expression data of the GSE7127 data set (*n*=63 melanoma cell lines) were downloaded as Affymetrix raw CEL files and processed as described above. Gene expression data of the GSE4843 data set (*n*=45 melanoma cell lines) were downloaded from GEO as series matrix file format that was already processed by authors of that respective study using the MAS 5.0 algorithm. Expression values were log_2_ transformed for further analysis such as heatmap cluster analysis and Pearson's correlation analysis.

### Gene set enrichment analysis

GSEA for transcription factor target gene sets negatively correlating with MITF expression levels in human melanoma cell line panels was performed using the BROAD javaGSEA standalone version (http://www.broadinstitute.org/gsea/downloads.jsp) and the curated C3 transcription factor target gene set collection (BROAD molecular signature database, MSigDbv4.0, http://www.broadinstitute.org/gsea/msigdb/index.jsp). We used the default setting of 1,000 permutations and the gene-set permutation mode. Gene probe identifiers were collapsed to symbols before the analysis. The probe ‘207233_s_at' representing *MITF* was used for Pearson's correlation analysis. For GSEA of siNT- versus siMITF-treated MZ7 melanoma cells, we used the mean of the log_2_ fold changes of two biological replicates as metric for the pre-ranked gene list algorithm (GSEAPreranked) of the BROAD javaGSEA tool with 1,000 permutation and the canonical pathway (cp) subcollection of the C2 curated BROAD molecular signature gene-set collection.

### Gene expression analysis of human melanoma cohorts

Gene expression data (RNA-seq) of the TGCA SKCM cohort was accessed through the cBioportal for Cancer Genomics (http://www.cbioportal.org) using the R-based package CGDS-R and following the TCGA guidelines for the use of TCGA data. We retrieved individual gene expression values for the genes of interest as RPKM (reads per kilobase of transcript per million mapped reads) normalized read counts. RPKM values <1 were set to 1, to avoid negative expression values on log_2_ tranformation if necessary. All melanoma samples were ordered by increasing *MITF* expression values. The expression of the TNF response gene signature was determined by calculating the mean expression per sample from all genes of the TNF response signature. The moving average of the gene expression was calculated using a sample window size of *n*=20 and added to the chart. Log_2_-normalized gene expression data from the Lund melanoma cohort (GSE65904) for candidate genes was used to generate moving average plots as described above.

### Analysis of published 3HA–MITF ChIP-seq data

The respective tracks including input and 3HA–MITF ChIP-seq results (GSE61967) were loaded into the UCSC genome browser. Significant 3HA–MITF binding peaks in the genomic region of the *JUN* locus were identified by statistical methods as previously described^35^.

### Statistical tests

All statistical tests were performed with the R-computing platform. We specify within the manuscript or legends the name of the statistical test, whether it is based on parametric or non-parametric data, its direction (one-sided or two-sided) and paired or unpaired. If applicable, we also performed corrections for multiple comparison as indicated in the figure legends using the respective methods.

## Additional information

**How to cite this article:** Riesenberg, S. *et al*. MITF and c-Jun antagonism interconnects melanoma dedifferentiation with pro-inflammatory cytokine responsiveness and myeloid cell recruitment. *Nat. Commun*. 6:8755 doi: 10.1038/ncomms9755 (2015).

## Supplementary Material

Supplementary Information Supplementary Figures 1-13 and Supplementary Tables 1-3

Supplementary Data 1 Gene symbol list of the TNF response gene set determined from human melanoma cell line panel (GSE51221) treated with TNF-alpha (72 hours) or left untreated.

Supplementary Data 2 List of genes (gene probes) and Pearson correlation values negatively correlating with the TNF response gene signature in the BROAD melanoma cell line panel.

Supplementary Data 3 Gene symbol list of manually selected bona fide pigmentation genes.

Supplementary Data 4 Non-mean centred log2 gene expression values (mean of two biological replicates) of genes from cluster 1 (induced by TNF) shown in figures 2c-e.

Supplementary Data 5 Non-mean centred log2 gene expression values (mean of two biological replicates) of genes from cluster 2 (induced by TNF) shown in figures 2c-e.

Supplementary Data 6 Log2 gene expression values (mean of two biological replicates) and log2 fold changes of siMITF versus siNT treated MZ7 melanoma cells.

Supplementary Data 7 GSEA results (Gene sets downregulated by MITF loss) from pre-ranked gene list mode analysis of siMITF treated versus siNT treated MZ7 melanoma cells. Log2 fold-change (siMITF-siNT) was used as metric for the analysis (see Supplementary Data 6).

Supplementary Software 1 R source codes
