# Supplementary material for: MITF and c-Jun antagonism interconnects melanoma dedifferentiation with pro-inflammatory cytokine responsiveness and myeloid cell recruitment
Source: Nat Commun. 2015 Nov 4;6:8755. doi: 10.1038/ncomms9755 (PMC4659938; doi:10.1038/ncomms9755)
Supplement: Supplementary Information — Supplementary Figures 1-13 and Supplementary Tables 1-3 [file ncomms9755-s1.pdf]

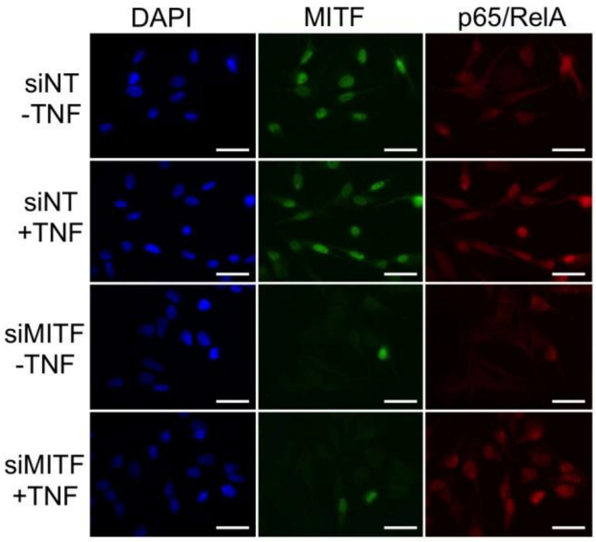

Supplementary Figure 1. Short-term depletion of MITF by RNAi has no effect on nuclear p65/RelA. (Related to main figure 2)

Representative immunofluorescent stainings of p65/RelA and MITF in siMITF treated MZ7 cells or non-targeting siRNA (siNT) treated controls. Experiment was repeated at least three times. Nuclei were counterstained with DAPI. Short-term TNF treatment (1000U ml^{-1}, 20min) served as positive control for p65/RelA nuclear translocation. Identical exposure times were used for the immunofluorescent images of DAPI, MITF or p65/RelA stains, respectively. Scale bars: 20µm.

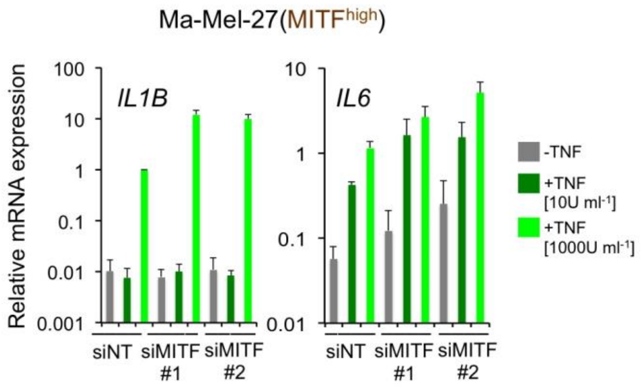

Supplementary Figure 2. Hyperresponsiveness to TNF-α in Ma.Mel27 cells by knockdown of MITF. (Related to main figure 2)

Analysis of relative mRNA expression levels of *IL1B* and *IL6* by qRT-PCR normalized to UBC on a logarithmic scale. Ma.Mel27 cells were treated with different concentrations of TNF for 24 hours or left untreated and transfected with the indicated siRNAs. Error bars indicate standard deviations from biological triplicates.

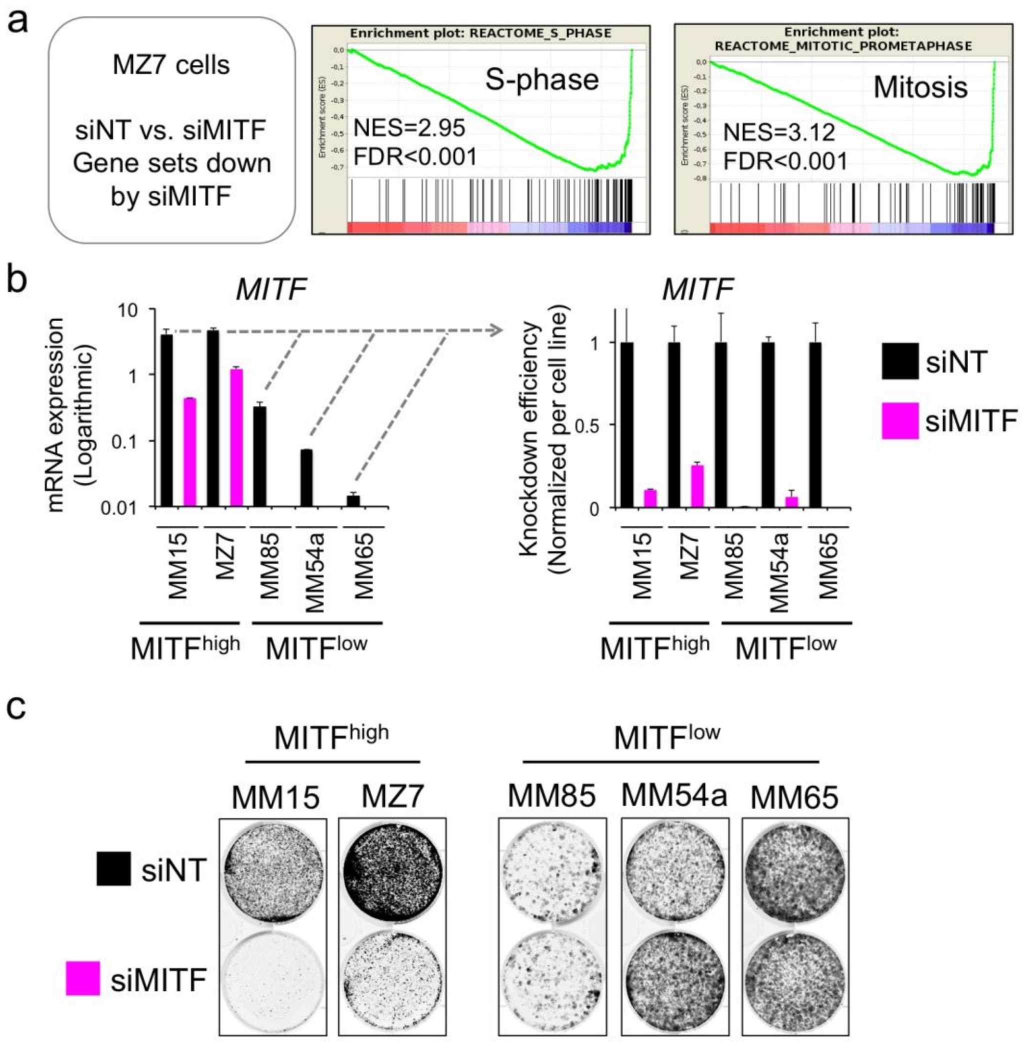

Supplementary Figure 3. Residual MITF levels are dispensable for proliferation of MITF^{low} melanoma cell lines. (Related to main figure 3)

(a) GSEA of siNT or siMITF treated MZ7 cells. Gene set enrichment plots of two top ranking gene sets related cell cycle (NES: Normalized Enrichment Score; FDR: False Discovery Rate). **(b)** MITF mRNA expression levels in MITF^{low} and MITF^{high} melanoma cell lines transfected with siNT or siMITF determined by qRT-PCR. Error bars indicate standard deviations of technical replicates from a representative experiment. Experiments were repeated three times. **(c)** Representative pictures of colony formation assays of siNT or siMITF transfected melanoma cell lines as indicated. Images show fixed culture dishes stained with crystal violet. Experiments were performed in duplicates and repeated three times.

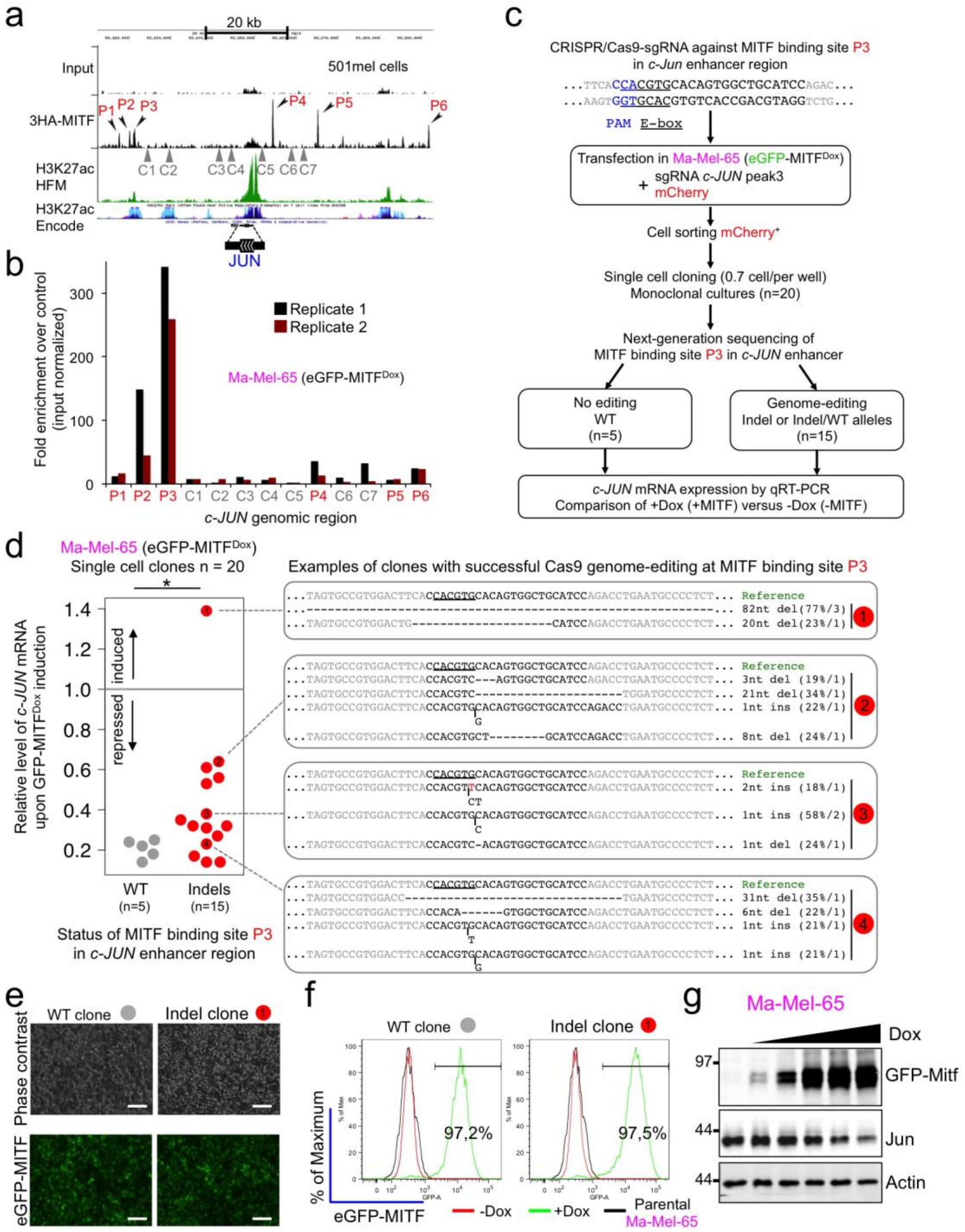

Supplementary Figure 4. Binding of MITF to enhancer region in the c-Jun locus contributes to c-Jun repression (Related to main figure 4). (a) Genome-browser view of 3xHA-MITF ChIP-seq profile from 501mel cells in the *c-JUN* region. Significant MITF binding peaks are indicated by black arrowheads and numbered by P1-P6 (red). Additional tracks show enhancer regions in human melanocytes and from the ENCODE project. Position of control regions for ChIP-qPCR validation are indicated by grey arrowheads and numbered from C1-C7 (grey). **(b)** Validation of MITF binding in Ma-Mel-65 ($MITF^{low}$) cells by ChIP-qPCR using conditional (Dox) expression of eGFP-MITF. ChIP for eGFP with GFP-trap comparing eGFP-MITF-expressing versus non-expressing cells. ChIP results are normalized to both input and enrichment of eGFP-MITF-expressing over non-expressing. Results from two biological replicates are shown (each performed as technical duplicate). **(c)** Outline of CRISPR-Cas9-based genome editing strategy to disrupt the genomic MITF-binding site within the major MITF-binding site P3 in Ma-Mel-65 cells. **(d)** Left panel: Relative suppression of *c-JUN* mRNA levels upon conditional expression of eGFP-MITF in an unbiased series (n=20) of Ma-Mel-65 single cell clones grouped by the genome-editing status (indels) at the major MITF-binding site P3. Four representative single cell clones with a mutated MITF-binding site P3 are highlighted by numbers. *p<0.05 (unpaired two-sided t-test). Right panels: Exemplary results from the genomic characterization by next-generation sequencing. Indel size and position relative to reference WT sequence are indicated (see also Supplementary Figure 5). Their relative representation by sequencing reads is given at the right. The sequencing results suggest tetraploidy of Ma-Mel-65 cells at this region and the presumed allelic distribution of the genome-editing events is indicated as integers. **(e-f)** Representative verification of equal eGFP-MITF expression in P3-WT and P3-Indel single cell clones by live-cell fluorescent images and FACS. Scale bars: 50µm. **(g)** Western blot showing dose-dependent suppression of c-Jun protein by eGFP-MITF in Ma-Mel-65 cells.

Supplementary Figure 5.

Summary of sequencing results form MITF-binding region P3 in 20 unbiased CRISPR-Cas9-genome engineered Ma-Mel65 (eGFP-MITF^{Dox}) single cell clones. Indel size and sequencing coverage are indicated. **(Related to Main Figure 4 and Supplementary Figure 4)**

Ma-Mel-65 clones <u>without</u> genome editing (WT, wild-type) at MITF-binding site P3.

E-box: <u>CACGTG</u>

WT#1
```
TAGTGCCGTGGACTTCACCACGTGCACAGTGGCTGCATCCAGACCTGAATGCCCCTCTCATTACTCACCGGAAGTGAGCT          REFERENCE Sequence

TAGTGCCGTGGACTTCACCACGTGCACAGTGGCTGCATCCAGACCTGAATGCCCCTCTCATTACTCACCGGAAGTGAGCT          WT        100%/1013 reads
```
WT#2
```
TAGTGCCGTGGACTTCACCACGTGCACAGTGGCTGCATCCAGACCTGAATGCCCCTCTCATTACTCACCGGAAGTGAGCT          REFERENCE Sequence

TAGTGCCGTGGACTTCACCACGTGCACAGTGGCTGCATCCAGACCTGAATGCCCCTCTCATTACTCACCGGAAGTGAGCT          WT        100%/1029 reads
```
WT#3
```
TAGTGCCGTGGACTTCACCACGTGCACAGTGGCTGCATCCAGACCTGAATGCCCCTCTCATTACTCACCGGAAGTGAGCT          REFERENCE Sequence

TAGTGCCGTGGACTTCACCACGTGCACAGTGGCTGCATCCAGACCTGAATGCCCCTCTCATTACTCACCGGAAGTGAGCT          WT        100%/827 reads
```
WT#4
```
TAGTGCCGTGGACTTCACCACGTGCACAGTGGCTGCATCCAGACCTGAATGCCCCTCTCATTACTCACCGGAAGTGAGCT          REFERENCE Sequence

TAGTGCCGTGGACTTCACCACGTGCACAGTGGCTGCATCCAGACCTGAATGCCCCTCTCATTACTCACCGGAAGTGAGCT          WT        100%/1059 reads
```
WT#5 **(scored as WT because of predominantly WT alleles)**
```
TAGTGCCGTGGACTTCACCACGTGCACAGTGGCTGCATCCAGACCTGAATGCCCCTCTCATTACTCACCGGAAGTGAGCT          REFERENCE Sequence

TAGTGCCGTGGACTTCACCACGTGCACAGTGGCTGCATCCAGACCTGAATGCCCCTCTCATTACTCACCGGAAGTGAGCT          WT        81%/523 reads
TAGTGCCGTGGACTTCACCACGTGCT--------GCATCCAGACCTGAATGCCCCTCTCATTACTCACCGGAAGTGAGCT          8nt del   19%/123 reads
```

Ma-Mel-65 clones <u>with successful</u> genome editing (Indels) at MITF-binding site P3.

E-box: <u>CACGTG</u>

Indel#1 **(shown in supplementary figure 4d)**
```
TAGTGCCGTGGACTTCACCACGTGCACAGTGGCTGCATCCAGACCTGAATGCCCCTCTCATTACTCACCGGAAGTGAGCT          REFERENCE Sequence

--------------------------------------------------------------------------------          82nt del  77%/1093 reads
TAGTGCCGTGGACTG--------------------CATCCAGACCTGAATGCCCCTCTCATTACTCACCGGAAGTGAGCT          20nt del  23%/320 reads
```

Indel#2 **(shown in supplementary figure 4d)**
```
TAGTGCCGTGGACTTCACCACGTGCACAGTGGCTGCATCCAGACCTGAATGCCCCTCTCATTACTCACCGGAAGTGAGCT          REFERENCE Sequence

TAGTGCCGTGGACTTCACCACGTC---AGTGGCTGCATCCAGACCTGAATGCCCCTCTCATTACTCACCGGAAGTGAGCT          3nt del   19%/171 reads
TAGTGCCGTGGACTTCACCACGTC---------------------TGGATGCCCCTCTCATTACTCACCGGAAGTGAGCT          21nt del  34%/303 reads
TAGTGCCGTGGACTTCACCACGTGCACAGTGGCTGCATCCAGACCTGAATGCCCCTCTCATTACTCACCGGAAGTGAGCT          1nt ins   22%/196 reads
                        |G
TAGTGCCGTGGACTTCACCACGTGCT--------GCATCCAGACCTGAATGCCCCTCTCATTACTCACCGGAAGTGAGCT          8nt del   24%/216 reads
```

Indel#3 **(shown in supplementary figure 4d)**
```
TAGTGCCGTGGACTTCACCACGTGCACAGTGGCTGCATCCAGACCTGAATGCCCCTCTCATTACTCACCGGAAGTGAGCT          REFERENCE Sequence

TAGTGCCGTGGACTTCACCACGTTCACAGTGGCTGCATCCAGACCTGAATGCCCCTCTCATTACTCACCGGAAGTGAGCT          2nt ins   18%/170 reads
                       |CT
TAGTGCCGTGGACTTCACCACGTGCACAGTGGCTGCATCCAGACCTGAATGCCCCTCTCATTACTCACCGGAAGTGAGCT          1nt ins   58%/538 reads
                       |C
TAGTGCCGTGGACTTCACCACGTC-ACAGTGGCTGCATCCAGACCTGAATGCCCCTCTCATTACTCACCGGAAGTGAGCT          1nt del   24%/218 reads
```

Indel#4 **(shown in supplementary figure 4d)**
```
TAGTGCCGTGGACTTCACCACGTGCACAGTGGCTGCATCCAGACCTGAATGCCCCTCTCATTACTCACCGGAAGTGAGCT          REFERENCE Sequence

TAGTGCCGTGGACC-------------------------------TGAATGCCCCTCTCATTACTCACCGGAAGTGAGCT          31nt del  35%/237 reads
TAGTGCCGTGGACTTCACCACA------GTGGCTGCATCCAGACCTGAATGCCCCTCTCATTACTCACCGGAAGTGAGCT          6nt del   22%/147 reads
TAGTGCCGTGGACTTCACCACGTGCACAGTGGCTGCATCCAGACCTGAATGCCCCTCTCATTACTCACCGGAAGTGAGCT          1nt ins   21%/143 reads
                       |T
TAGTGCCGTGGACTTCACCACGTGCACAGTGGCTGCATCCAGACCTGAATGCCCCTCTCATTACTCACCGGAAGTGAGCT          1nt ins   21%/141 reads
                        |G
```

Indel#5
```
TAGTGCCGTGGACTTCACCACGTGCACAGTGGCTGCATCCAGACCTGAATGCCCCTCTCATTACTCACCGGAAGTGAGCT          REFERENCE Sequence

TAGTGCCGTGGACTTCAG------------------------ACCTGAATGCCCCTCTCATTACTCACCGGAAGTGAGCT          24nt del  48%/474 reads
TAGTGCCGTGGACTTCACG-----CACAGTGGCTGCATCCAGACCTGAATGCCCCTCTCATTACTCACCGGAAGTGAGCT          5nt del   34%/333 reads
TAGTGCCGTGGACTTCACCACGTA--CAGTGGCTGCATCCAGACCTGAATGCCCCTCTCATTACTCACCGGAAGTGAGCT          2nt del   18%/177 reads
```

Indel#6
```
TAGTGCCGTGGACTTCACCACGTGCACAGTGGCTGCATCCAGACCTGAATGCCCCTCTCATTACTCACCGGAAGTGAGCT          REFERENCE Sequence

-----------------------CCACAGTGGCTGCATCCAGACCTGAATGCCCCTCTCATTACTCACCGGAAGTGAGCT          23nt del  32%/250 reads
TAGTGCCGTGGACTTCACCACGTGCACAGTGGCTGCATCCAGACCTGAATGCCCCTCTCATTACTCACCGGAAGTGAGCT          2nt ins   14%/109 reads
                       |TC
TAGTGCCGTGGACTTCACCACGTC-ACAGTGGCTGCATCCAGACCTGAATGCCCCTCTCATTACTCACCGGAAGTGAGCT          1nt del   25%/198 reads
TAGTGCCGTGGACTTCACCACGTGCACAGTGGCTGCATCCAGACCTGAATGCCCCTCTCATTACTCACCGGAAGTGAGCT          1nt ins   29%/229 reads
                        |G
```

Supplementary Figure 5 (continued).

Summary of sequencing results form MITF-binding region P3 in 20 unbiased CRISPR-Cas9-genome engineered Ma-Mel65 (eGFP-MITF^{Dox}) single cell clones. Indel size and sequencing coverage are indicated.

Indel#7 (likely presence of second clone at low frequency)

```
TAGTGCCGTGGACTTCACCACGTGCACAGTGGCTGCATCCAGACCTGAATGCCCCTCTCATTACTCACCGGAAGTGAGCT          REFERENCE Sequence

TAGTGCCGTGGACG----------CACAGTGGCTGCATCCAGACCTGAATGCCCCTCTCATTACTCACCGGAAGTGAGCT          10nt del  33%/310 reads
TAGTGCCGTGGACTG--------------------CATCCAGACCTGAATGCCCCTCTCATTACTCACCGGAAGTGAGCT          20nt del  44%(405 reads


TAGTGCCGTGGACTTCACCACGTGCACAGTGGCTGCATCCAGACCTGAATGCCCCTCTCATTACTCACCGGAAGTGAGCT          WT        16%/146 reads
TAGTGCCGTGGACTTCACCACGTGCACAGTGGCTGCATCCAGACCTGAATGCCCCTCTCATTACTCACCGGAAGTGAGCT          1nt ins   3%/25 reads
                       |T
TAGTGCCGTGGACTTCACCACGTC-ACAGTGGCTGCATCCAGACCTGAATGCCCCTCTCATTACTCACCGGAAGTGAGCT          1nt del   4%/40 reads
```

Indel#8

```
TAGTGCCGTGGACTTCACCACGTGCACAGTGGCTGCATCCAGACCTGAATGCCCCTCTCATTACTCACCGGAAGTGAGCT          REFERENCE Sequence

TAGTGCCGTGGG------------CACAGTGGCTGCATCCAGACCTGAATGCCCCTCTCATTACTCACCGGAAGTGAGCT          12nt del  27%/144 reads
TAGTGCCGTGGACTTCACCACA------GTGGCTGCATCCAGACCTGAATGCCCCTCTCATTACTCACCGGAAGTGAGCT          6nt del   56%/298 reads
TAGTGCCGTGGACTTCACCACGTA--CAGTGGCTGCATCCAGACCTGAATGCCCCTCTCATTACTCACCGGAAGTGAGCT          2nt del   18%/94 reads
```

Indel#9

```
TAGTGCCGTGGACTTCACCACGTGCACAGTGGCTGCATCCAGACCTGAATGCCCCTCTCATTACTCACCGGAAGTGAGCT          REFERENCE Sequence

TAGTGCCGTGGACC-------------------------------TGAATGCCCCTCTCATTACTCACCGGAAGTGAGCT          31nt del  46%/204 reads
TAGTGCCGTGGACTTCAT--------------------CCAGACCTGAATGCCCCTCTCATTACTCACCGGAAGTGAGCT          20nt del  17%/75 reads
TAGTGCCGTGGACTTCACCACGTGCACAGTGGCTGCATCCAGACCTGAATGCCCCTCTCATTACTCACCGGAAGTGAGCT          1nt ins   15%/67 reads
                      |T
TAGTGCCGTGGACTTCACCACGTGCACAGTGGCTGCATCCAGACCTGAATGCCCCTCTCATTACTCACCGGAAGTGAGCT          1nt ins   23%/101 reads
                        |G
```

Indel#10 (likely presence of second clone at low frequency)

```
TAGTGCCGTGGACTTCACCACGTGCACAGTGGCTGCATCCAGACCTGAATGCCCCTCTCATTACTCACCGGAAGTGAGCT          REFERENCE Sequence

TAGTGCCGTGGACTTCG-------CACAGTGGCTGCATCCAGACCTGAATGCCCCTCTCATTACTCACCGGAAGTGAGCT          7nt del   20%/168 reads
TAGTGCCGTGGACTTCACCAG---------------------ACCTGAATGCCCCTCTCATTACTCACCGGAAGTGAGCT          21nt del  30%/246 reads
TAGTGCCGTGGACTTCACCACGTGCACAGTGGCTGCATCCAGACCTGAATGCCCCTCTCATTACTCACCGGAAGTGAGCT          1nt ins   29%/236 reads
                       |A/T

TAGTGCCGTGGACTTCACCACGTC-ACAGTGGCTGCATCCAGACCTGAATGCCCCTCTCATTACTCACCGGAAGTGAGCT                    11%/95 reads
TAGTGCCGTGGACTTCACCACGTGCACAGTGGCTGCATCCAGACCTGAATGCCCCTCTCATTACTCACCGGAAGTGAGCT          WT        4%/32 reads
TAGTGCCA---------------GCACAGTGGCTGCATCCAGACCTGAATGCCCCTCTCATTACTCACCGGAAGTGAGCT          15nt del  3%/26 reads
TAGTGCCGTGGACTTCACCACGTGCACAGTGGCTGCATCCAGACCTGAATGCCCCTCTCATTACTCACCGGAAGTGAGCT          1nt ins   3%/24 reads
                        |G
```

Indel#11

```
TAGTGCCGTGGACTTCACCACGTGCACAGTGGCTGCATCCAGACCTGAATGCCCCTCTCATTACTCACCGGAAGTGAGCT          REFERENCE Sequence

TAGTGCCGTGA------------------------CATCCAGACCTGAATGCCCCTCTCATTACTCACCGGAAGTGAGCT          24nt del  28%/232 reads
TAGTGCCGTGGACTTCACCAG---CACAGTGGCTGCATCCAGACCTGAATGCCCCTCTCATTACTCACCGGAAGTGAGCT          3nt del   18%/146 reads
TAGTGCCGTGGACTTCACCAT-----------------CCAGACCTGAATGCCCCTCTCATTACTCACCGGAAGTGAGCT          17nt del  24%/196 reads
TAGTGCCGTGGACTTCACCACA------GTGGCTGCATCCAGACCTGAATGCACAGTGGCTGCATCCAGACCTGAATGCC*         6nt del   19%/155 reads
TAGTGCCGTGGACTTCACCACGTGCACAGTGGCTGCATCCAGACCTGAATGCCCCTCTCATTACTCACCGGAAGTGAGCT          1nt ins   12%/104 reads
                       |T
```

Indel#12

```
TAGTGCCGTGGACTTCACCACGTGCACAGTGGCTGCATCCAGACCTGAATGCCCCTCTCATTACTCACCGGAAGTGAGCT          REFERENCE Sequence

TAGTGCCGTGGACTTCACCACGTGCACAGTGGCTGCATCCAGACCTGAATGCCCCTCTCATTACTCACCGGAAGTGAGCT          WT        81% (523 reads)
TAGTGCCGTGGACTTCACCACGTGCT--------GCATCCAGACCTGAATGCCCCTCTCATTACTCACCGGAAGTGAGCT          8nt del   19% (123 reads)
```

Indel#13

```
TAGTGCCGTGGACTTCACCACGTGCACAGTGGCTGCATCCAGACCTGAATGCCCCTCTCATTACTCACCGGAAGTGAGCT          REFERENCE Sequence

TAGTGCCGTGGACTTCACCACGC--ACAGTGGCTGCATCCAGACCTGAATGCCCCTCTCATTACTCACCGGAAGTGAGCT          2nt del   28%/117 reads
TAGTGCCGTGGACTTCACCACGTGCACAGTGGCTGCATCCAGACCTGAATGCCCCTCTCATTACTCACCGGAAGTGAGCT          1nt ins   13%/52 reads
                       |A
TAGTGCCGTGGACTTCACCACGTGCACAGTGGCTGCATCCAGACCTGAATGCCCCTCTCATTACTCACCGGAAGTGAGCT          1nt ins   59%/243 reads
                        |G
```

Indel#14

```
TAGTGCCGTGGACTTCACCACGTGCACAGTGGCTGCATCCAGACCTGAATGCCCCTCTCATTACTCACCGGAAGTGAGCT          REFERENCE Sequence

TAGTGCCGTGGC---------------------TGCATCCAGACCTGAATGCCCCTCTCATTACTCACCGGAAGTGAGCT          21nt del  38% (327 reads)
TAGTGCCGTGGACTTCACCACGC--ACAGTGGCTGCATCCAGACCTGAATGCCCCTCTCATTACTCACCGGAAGTGAGCT          2nt del   42% (360 reads)
TAGTGCCGTGGACTTCACCACGTGCACAGTGGCTGCATCCAGACCTGAATGCCCCTCTCATTACTCACCGGAAGTGAGCT          3nt ins   18% (157 reads)
                       |TAT
```

Indel#15

```
TAGTGCCGTGGACTTCACCACGTGCACAGTGGCTGCATCCAGACCTGAATGCCCCTCTCATTACTCACCGGAAGTGAGCT          REFERENCE Sequence

TAGTGCCGTGGACTG--------------------CATCCAGACCTGAATGCCCCTCTCATTACTCACCGGAAGTGAGCT          20nt del  31%/245 reads
TAGTGCCGTGGACTTCACA---------GTGGCTGCATCCAGACCTGAATGCCCCTCTCATTACTCACCGGAAGTGAGCT          9nt del   23%/182 reads
TAGTGCCGTGGACTTCACCACGG---CAGTGGCTGCATCCAGACCTGAATGCCCCTCTCATTACTCACCGGAAGTGAGCT          3nt del   26%/202 reads
TAGTGCCGTGGACTTCACCACGTGCACAGTGGCTGCATCCAGACCTGAATGCCCCTCTCATTACTCACCGGAAGTGAGCT          1nt ins   20%/154 reads
                        |A
```

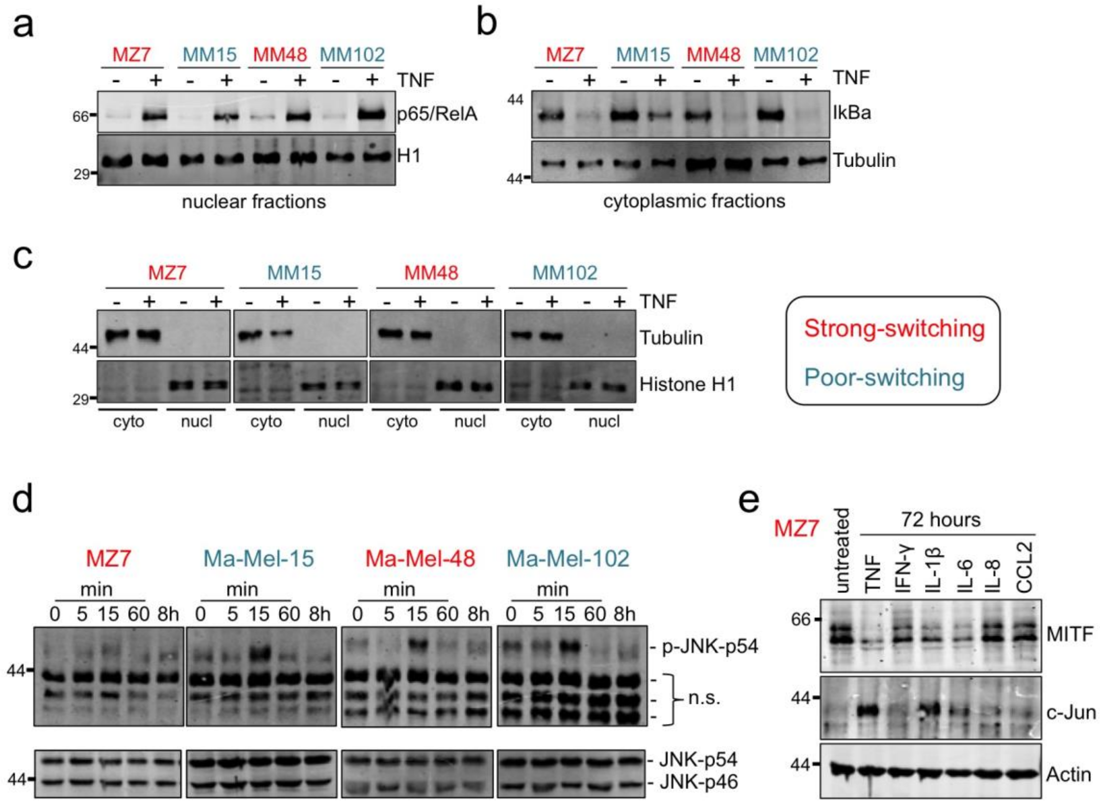

Supplementary Figure 6. TNF signalling in strong- and poor-switching human melanoma cell lines and impact of different cytokines on dedifferentiation. (Related to main figure 5)

(a) Immunoblot for p65/RelA in nuclear fractions of TNF-treated (1000U ml^{-1}, 20min) or untreated melanoma cell lines. The nuclear protein histone H1 (H1) is shown as loading control. **(b)** Expression of IkBa cytoplasmic fractions of TNF-treated (1000U ml^{-1}, 20min) or untreated melanoma cell lines. Levels of cytoplasmic tubulin are shown as loading control. **(c)** Immunoblots for tubulin and Histone H1 confirm successful separation of cytoplasmic and nuclear fractions from the same lysates as shown in a-b. **(d)** Time course experiment of TNF stimulation (1000U ml^{-1}) showing transient phosphorylation of JNK. Levels of total JNK verify equal loading. Prominent non-specific (n.s.) bands mask the band corresponding to phosphorylated p-JNK-p46. **(e)** Immunoblot for MITF and c-Jun in MZ7 melanoma cell lines treated with the indicated cytokines for 72 hours (CCL2 100ng ml^{-1}, IFN-gamma 1000U ml^{-1}, IL-1b 1000U ml^{-1}, IL-6 1000U ml^{-1}, IL-8 150ng ml^{-1}, TNF-alpha 1000U ml^{-1}). Actin levels verify equal loading of the samples.

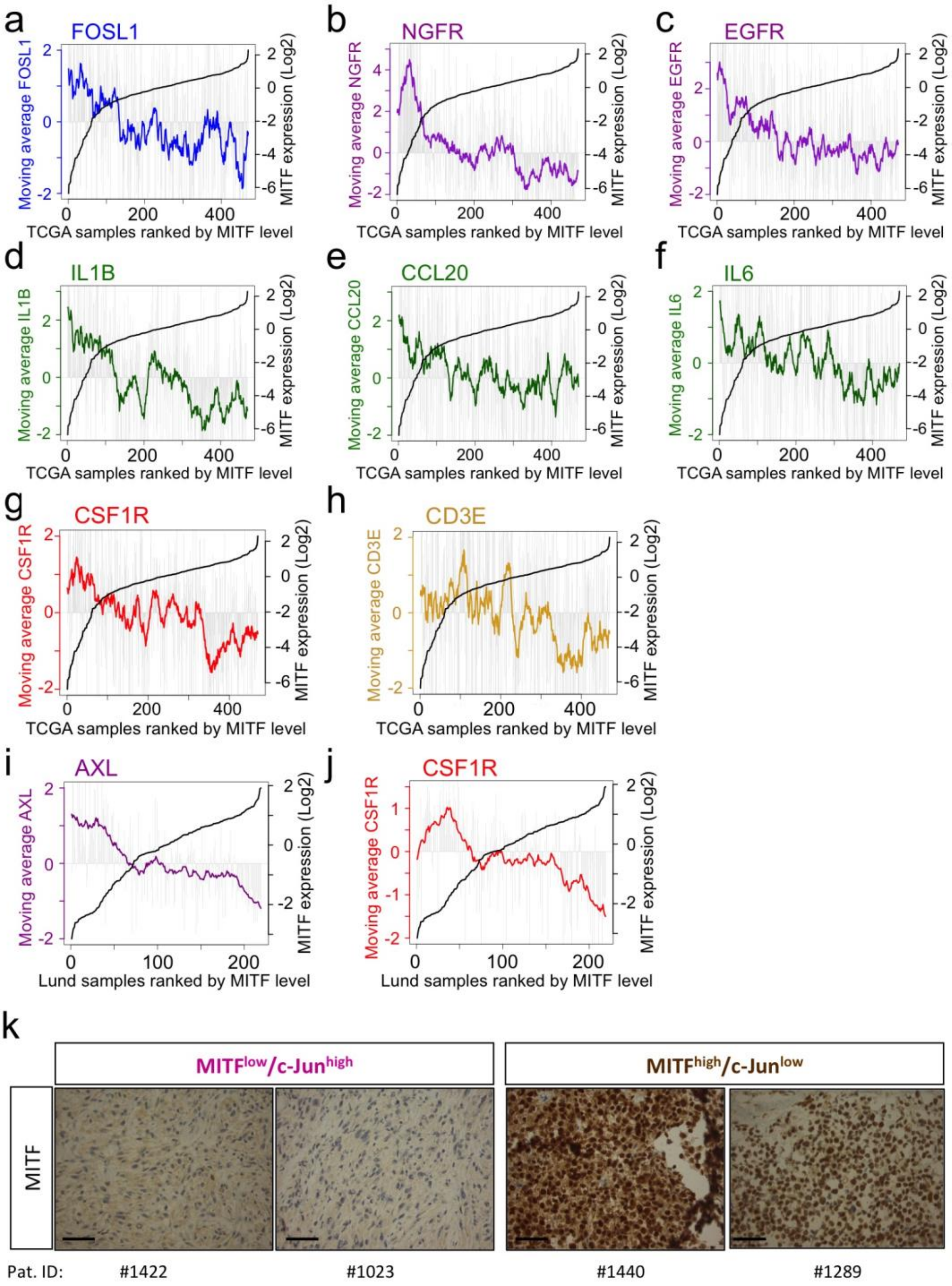

Supplementary Figure 7. Expression of cell state, cytokine and immune cell marker genes in the TCGA and Lund melanoma cohort. (Related to main figure 6)

(a-h) Expression of *FOSL1*, *NGFR*, *EGFR* (dedifferentiated cell state); *IL1B*, *CCL20*, *IL6* (cytokine); *CSF1R* (myeloid), *CD3E* (T-cell) in TCGA melanoma samples ordered by increasing *MITF* levels from left to right in each panel. The black line (corresponding y-axis at the right side of each panel) shows the increasing MITF expression levels. Grey bars represent the respective expression levels of *FOSL1*, *NGFR*, *EGFR, IL1B*, *CCL20*, *IL6, CSF1R* or *CD3E* in each individual sample. The coloured lines reflect the expression trends as determined by a moving average algorithm with a sample window size of n=20. **(i-j)** Expression of *AXL*, *CSF1R* in the Lund melanoma samples relative to increasing *MITF* level. Moving average plot was done as described in a-h. (k) Immunohistochemical stain for MITF in representative Lund melanoma cohort specimens. Tissue section plains differ from the stains shown in the main figure 6. Scale bars: 50µm.

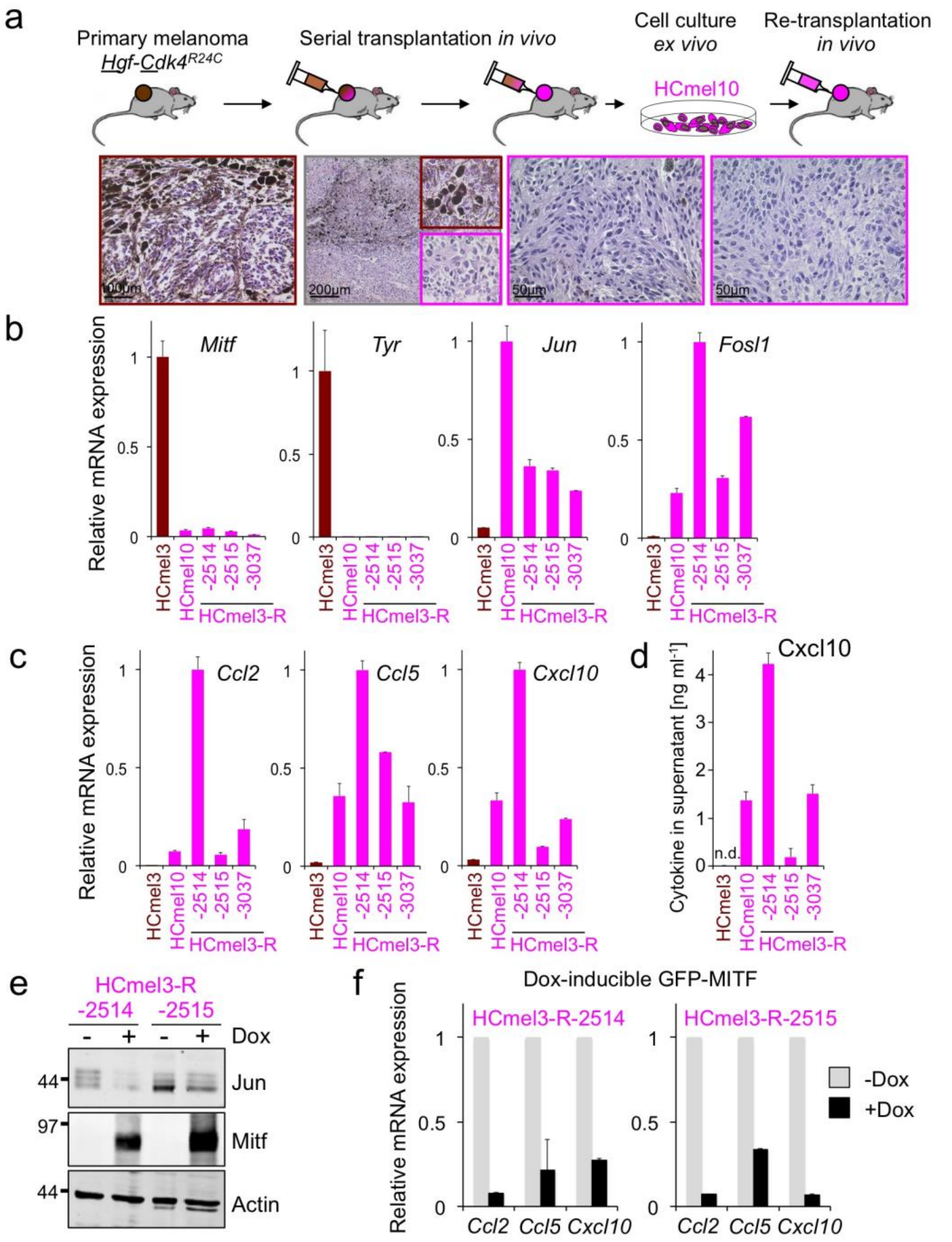

Supplementary Figure 8. Establishment of the HCmel10 cell line and characterization of MITF^{low}/c-Jun^{high} inflammatory cell state in HCmel3-R and HCmel10 mouse melanoma cell lines (Related to main figure 7).

(a) Cartoon showing the generation of the HCmel10 cell line by serial *in vivo* transplantation and histological documentation of progressive dedifferentiation. Scale bars are either 100µm, 200µm or 50µm as indicated in the respective panels. **(b)** Expression of *Mitf*, *Tyr*, *c-Jun* and *Fosl1* in HCmel3, HCmel3-R and HCmel10 mouse melanoma cell lines by qRT-PCR. Error bars indicate standard deviations from biological triplicates. **(c)** Expression of *Ccl2*, *Ccl5* and *Cxcl10* in HCmel3, HCmel3-R and HCmel10 mouse melanoma cell lines by qRT-PCR. Error bars indicate standard deviations from two biological triplicates. **(d)** Levels of secreted Cxcl10 in the supernatant of HCmel3, HCmel3-R and HCmel10 mouse melanoma cell lines determined by ELISA. Error bars indicate standard deviations from biological triplicates. **(e-f)** Conditional re-expression of Mitf (Dox-inducible eGFP-MITF) suppresses c-Jun protein level (by Western blot) and chemokine (*Ccl2*, *Ccl5*, *Cxcl10*) expression (by qRT-PCR). Error bars indicate standard deviations of technical replicates. This representative experiment was performed in parallel to the immunoblot shown in e. The Dox-stimulation was independently repeated three times.

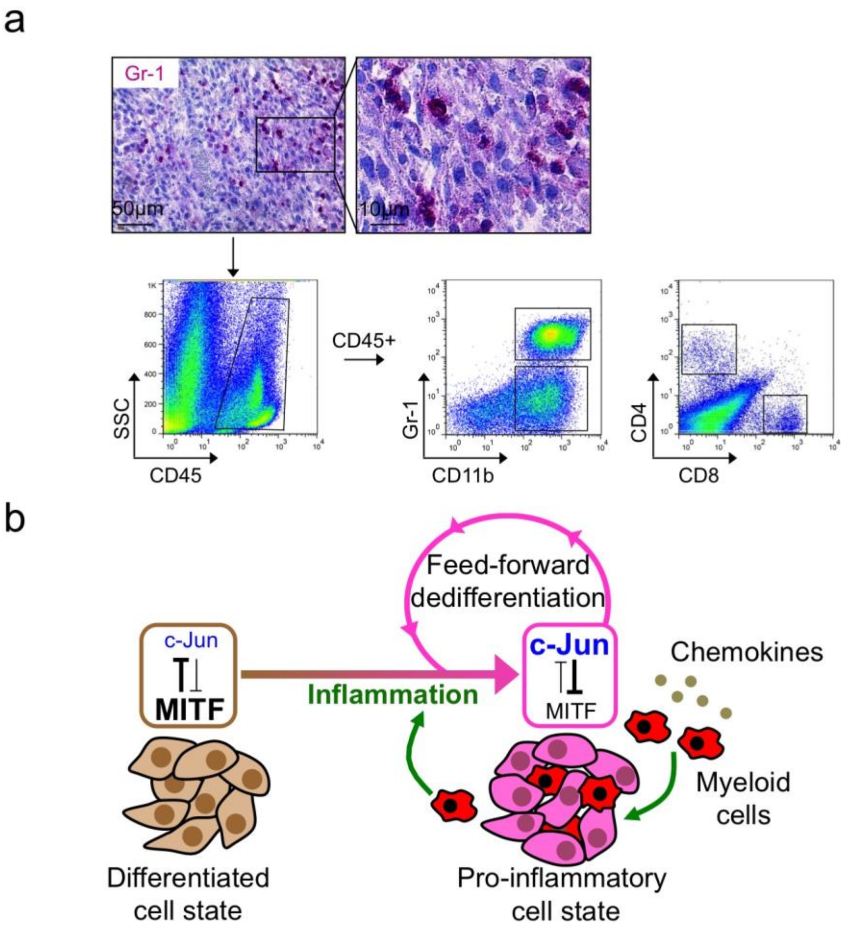

Supplementary Figure 9. FACS-gating strategy of immune cell analysis of syngeneic melanomas and model summarizing the findings of the work. (Related to main figure 7)
(a) Upper panels: Representative picture of a myeloid cell (Gr-1+)-rich HCmel10 syngeneic mouse melanoma (Low and high magnification view: scale bars 50µm and 10µm). Lower panels: Exemplary FACS-gating strategy identifying CD45+ immune cells followed by sub-specification using respective surface markers for myeloid cells (CD11b, Gr-1) and T-cells (CD4, CD8). **(b)** Model summarizing the findings of the study. Inflammatory signals cause melanoma cell dedifferentiation and the MITF/c-Jun antagonism initiates a feed-forward loop of progressive dedifferentiation that is linked to the acquisition of a pro-inflammatory cell state orchestrating myeloid cell recruitment.

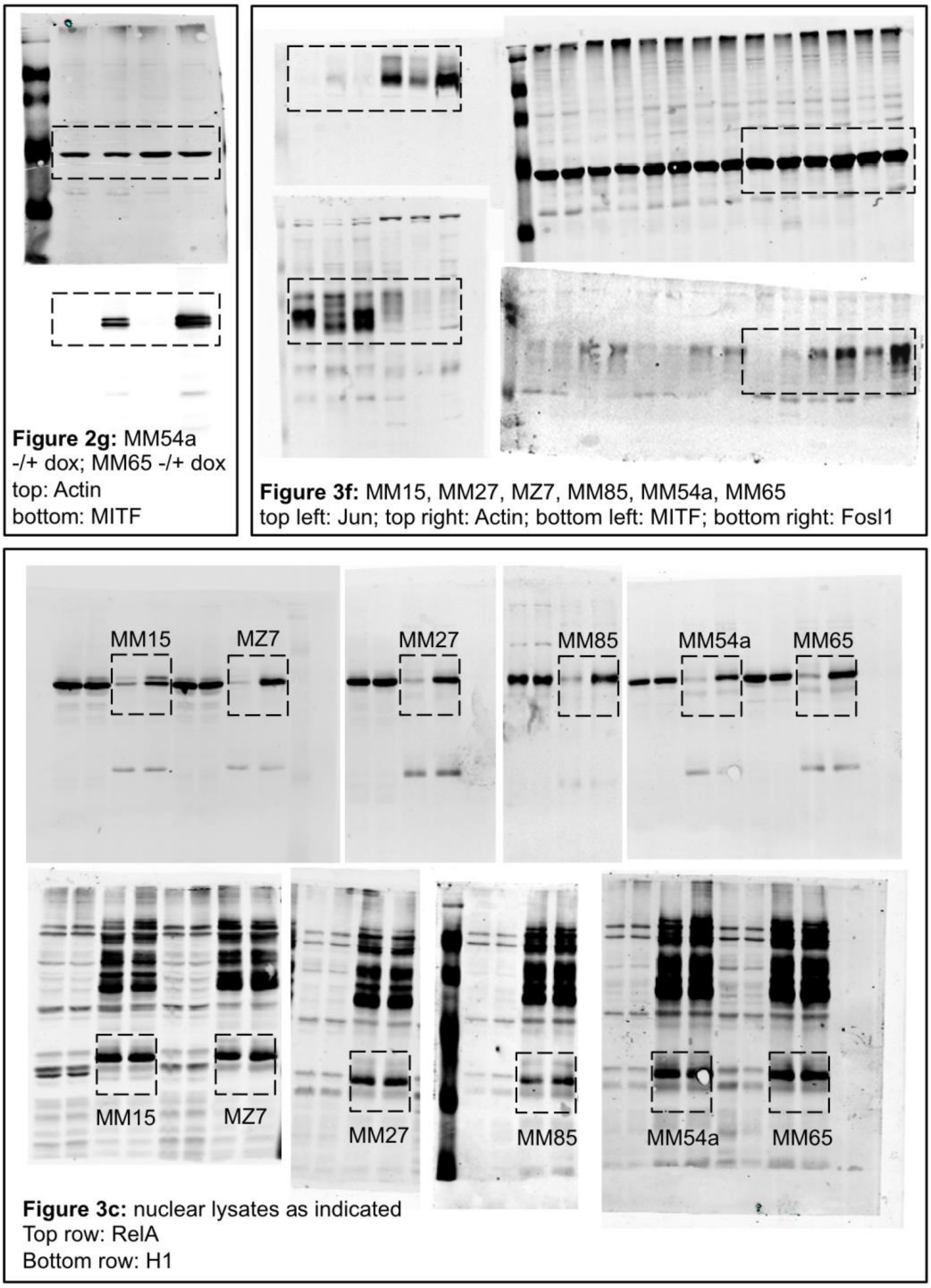

Supplementary Figure 10. Full blots of immunoblots shown in the main figures of the manuscript. The respective main figures are indicated.

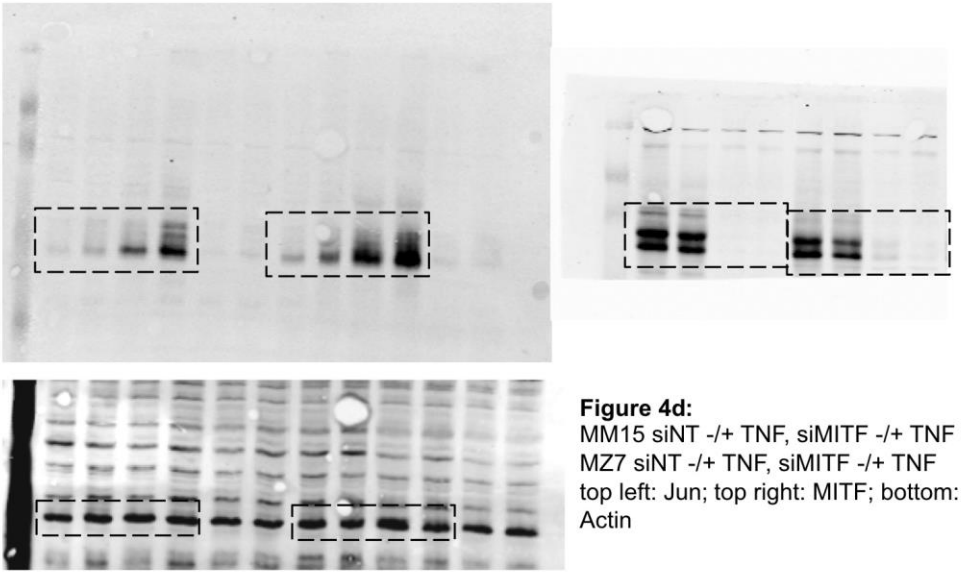

Figure 4d:
MM15 siNT -/+ TNF, siMITF -/+ TNF
MZ7 siNT -/+ TNF, siMITF -/+ TNF
top left: Jun; top right: MITF; bottom: Actin

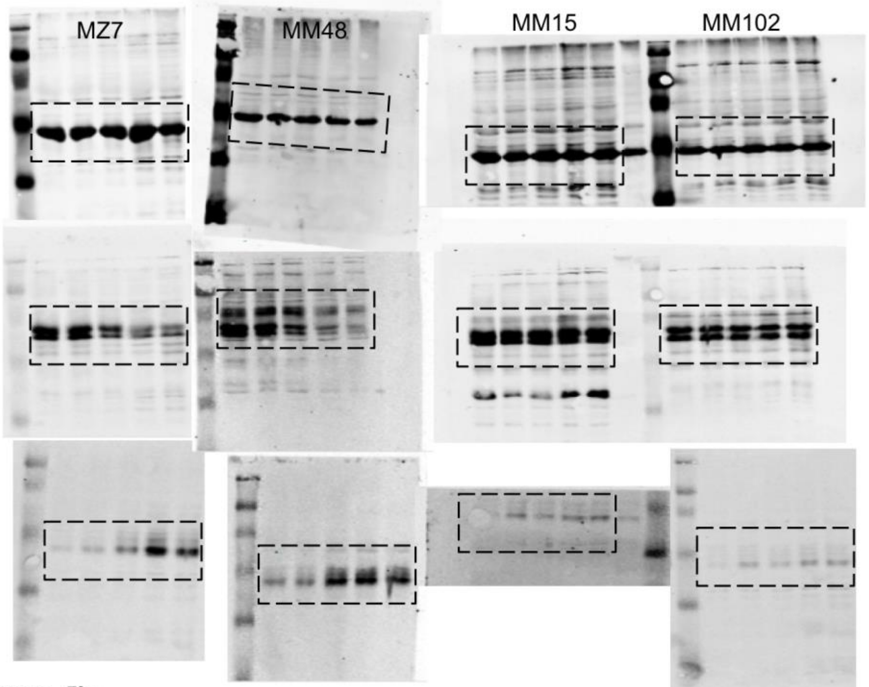

Figure 5b:
Cell lines as indicated, each treated with TNF for 0, 8, 24, 48, and 72h
Top row: Actin; middle: MITF; bottom: Jun

Supplementary Figure 11. Full blots of immunoblots shown in the main figures of the manuscript. The respective main figures are indicated.

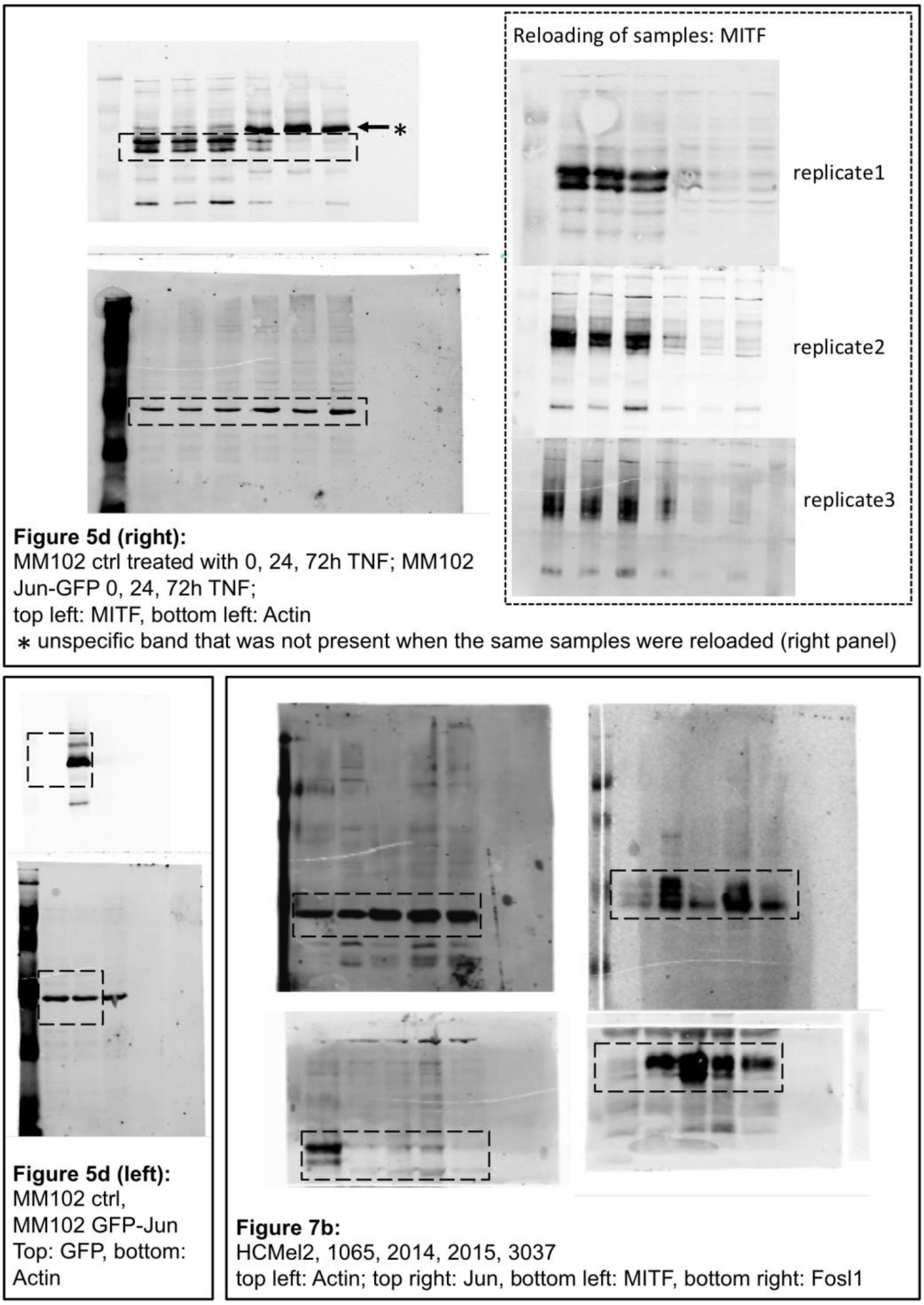

Supplementary Figure 12. Full blots of immunoblots shown in the main figures of the manuscript. The respective main figures are indicated.

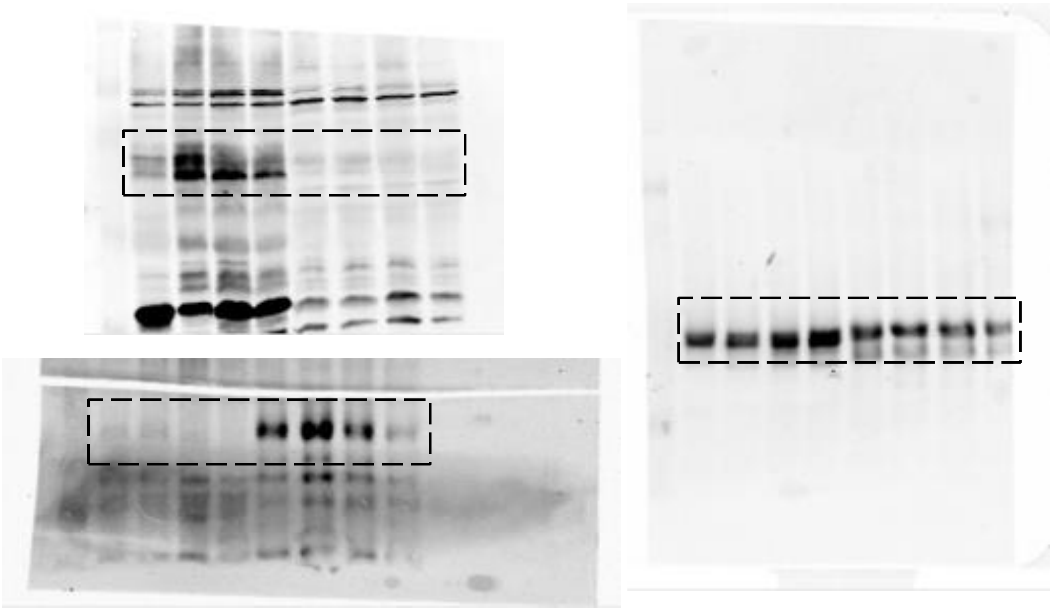

Figure 7f:
4x HCMel3 tumor lysates, 4x HCMel10 tumor lysates
Top left: Mitf; bottom left: Jun, right: ERK

Supplementary Figure 13. Full blots of immunoblots shown in the main figures of the manuscript. The respective main figures are indicated.

Supplementary Table 1. Human melanoma cell lines used in this study.

Molecular characterization

Name	Abbreviation	BRAF	NRAS	HRAS	Other driver mutations	MITFhigh / MITFlow by microarray
Ma-Mel-04	MM04	V600E	wt	wt		MITFhigh
Ma-Mel-15	MM15	wt	wt	wt		MITFhigh
Ma-Mel-19	MM19	V600E	wt	wt		MITFlow
Ma-Mel-27	MM27	wt	G12D	wt		MITFhigh
Ma-Mel-36	MM36	V600E	wt	wt		MITFhigh
Ma-Mel-37b	MM37b	wt	Q61L	wt		MITFhigh
Ma-Mel-48a	MM48a	G469R	wt	G13I		MITFhigh
Ma-Mel-54a	MM54a	V600E	wt	wt		MITFlow
Ma-Mel-65	MM65	wt	Q61K	wt	NF1 (R440*); KIT (V569A)	MITFlow
Ma-Mel-67	MM67	V600K	wt	wt	NF1 (N2602I)	MITFhigh
Ma-Mel-71	MM71	wt	wt	wt	NF1 (T2667fs)	MITFhigh
Ma-Mel-79b	MM79b	wt	Q61K	wt	NF1 (D1849Y)	MITFhigh
Ma-Mel-85	MM85	V600E	wt	wt		MITFlow
Ma-Mel-102a	MM102a	wt	Q61L	wt		MITFhigh
Ma-Mel-103b	MM103b	wt	Q22K	wt	NF1 (W784*)	MITFlow
MZ7-mel	MZ7	V600E	wt	wt		MITFhigh
SK-Mel-28	SK28	V600E	wt	wt		MITFhigh

Clinical parameters and references

Name	Age at biopsy (years)	Gender	Localization primary	Type	Stage (AJCC) at biopsy	Biopsy origin	Survival after biopsy (months)	References (PMIDs)
Ma-Mel-04	29	m	skin	n.a.	IV	urinary bladder	13	17311103; 22817889
Ma-Mel-15	39	f	occult	occult	IV	lymph node	1+ *	17311103; 22817889
Ma-Mel-19	62	f	skin	SSM	IV	cut./sc. metastasis§	0.2+ *	17311103; 22817889
Ma-Mel-27	58	f	skin	ALM	IV	cut./sc. metastasis§	7.5	17311103; 22817889
Ma-Mel-36	27	f	skin	n.a.	IV	cut./sc. metastasis§	4	17311103; 22817889
Ma-Mel-37b	59	f	skin	NM	III	lymph node	63.2+ *	17311103; 22817889
Ma-Mel-48a	87	m	skin	NM	IV	cut./sc. metastasis§	13.8	17311103; 22817889
Ma-Mel-54a	41	f	skin	SSM	IV	lymph node	3.7	17311103; 22817889
Ma-Mel-65	24	f	skin	SSM	III	lymph node	26.1+ *	17311103; 22817889
Ma-Mel-67	51	f	skin	SSM	IV	lymph node	26.7+ *	17311103; 22817889
Ma-Mel-71	46	m	skin	ALM	IV	cut./sc. metastasis§	4	17311103; 22817889
Ma-Mel-79b	41	m	skin	NM	IV	cut./sc. metastasis§	10.6+ *	17311103; 22817889
Ma-Mel-85	38	m	occult	occult	IV	cut./sc. metastasis§	3.3	17311103; 22817889
Ma-Mel-102a	74	m	skin	SSM	III	lymph node	10.4+ *	17311103; 22817889
Ma-Mel-103b	48	f	skin	NM	IV	cut./sc. metastasis§	4.8	17311103; 22817889
MZ7-mel	26	f	skin	n.a.	IV	splenic metastasis	56	16247014 (see table 2 in ref.)
SK-Mel-28	51	m			IV	lymph node		1067619; 327080; 21725359 #

§ cutaneous/subcutaneous metastasis

* longer follow-up not publicly available

#see also: https://www.mskcc.org/research-advantage/support/technology/tangible-material/human-melanoma-cell-line-sk-mel-28

Supplementary Table 2. Mouse melanoma cell lines used in this study.

Name	Genetic background	Source	Melanocytic differentiation status (in vitro)	Melanocytic differentiation status (in vivo)	Reference
HCmel3	Hgf-Cdk4R24C	Primary melanoma	differentiated	differentiated	PMID:23051752
HCmel3-R-2514	Hgf-Cdk4R24C	HCmel3 relapse from T-cell therapy	dedifferentiated	differentiated	PMID:23051752
HCmel3-R-2515	Hgf-Cdk4R24C	HCmel3 relapse from T-cell therapy	dedifferentiated	dedifferentiated	this study
HCmel3-R-3037	Hgf-Cdk4R24C	HCmel3 relapse from T-cell therapy (two consecutive rounds)	dedifferentiated	partially differentiated	this study
HCmel10	Hgf-Cdk4R24C	Serial transplant primary melanoma	dedifferentiated	dedifferentiated	this study

Supplementary Table 3.

List of all PCR primers, DNA oligos and siRNA sequences used in this study.

qRT-PCR primers (human genes)

**Symbol**	**Left primer 5'-3'**	**Right primer 5'-3'**
AXL	ACCTACTCTGGCTCCAGGATG	CGCAGGAGAAAGAGGATGTC
CCL20	GTGCTGCTACTCCACCTCTG	CGTGTGAAGCCCACAATAAA
IL1B	GAAGCTGATGGCCCTAAACA	AAGCCCTTGCTGTAGTGGTG
IL6	AGTGAGGAACAAGCCAGAGC	CATTTGTGGTTGGGTCAGG
IL8	CGGAAGGAACCATCTCACTG	AGCACTCCTTGGCAAAACTG
JUN	TCTCACAAACCTCCCTCCTG	GAGGGGGTTACAAACTGCAA
MITF	GAAATCTTGGGCTTGATGGA	AGGAGTTGCTGATGGTGAGG
MLANA	GCTCATCGGCTGTTGGTATT	TTCTTGTGGGCATCTTCTTG
NGFR	CTGCTGCTGTTGCTGCTTCT	CAGGCTTTGCAGCACTCAC
THBS1	CACAGCTCGTAGAACAGGAGG	CAATGCCACAGTTCCTGATG
UBC	GGAGCCGAGTGACACCATTG	CAGGGTACGACCATCTTCCAG

qRT-PCR primers (mouse genes)

**Symbol**	**Left primer 5'-3'**	**Right primer 5'-3'**
Ccl2	GGGATCATCTTGCTGGTGAA	AGGTCCCTGTCATGCTTCTG
Ccl5	GTGCCCACGTCAAGGAGTAT	CCACTTCTTCTCTGGGTTGG
Cxcl10	CTCATCCTGCTGGGTCTGAG	CCTATGGCCCTCATTCTCAC
Fosl1	CTCTTCCTCCTCTGGGCTG	ATCCCCAGTACAGTCCCCCT
Jun	GGGACACAGCTTTCACCCTA	GAAAAGTAGCCCCCAACCTC
Mitf	TCAAGTTTCCAGAGACGGGT	CATCATCAGCCTGGAATCAA
Tyr	ATAGGTGCATTGGCTTCTGG	TCTTCACCATGCTTTTGTGG
Ubc	AGGCAAGACCATCACCTTGGACG	CCATCACACCCAAGAACAAGCACA

ChIP-qPCR primers (mouse genes)

**Name**	**Left primer 5'-3'**	**Right primer 5'-3'**
Peak1 (P1)	GGATTTGGTACCAGACAAGGC	CTCCAACTCCTGACCACGT
Peak2 (P2)	CTGTGCTCCCATGGTCCTAG	ACCCTCCATAAGCACAGACC
Peak3 (P3)	CACAGACCTTTCTCGGGACT	CCAGTGCAACTATGGCTGTC
Control1 (C1)	AAACTCCGAGTCTGCTTCCA	AGTGTGGTTGTAGTGGGAGG
Control2 (C2)	AGCCAGTTTTGAAGAAGGCG	AGCCTCTCAAAGTCACGGAA
Control3 (C3)	CAGGGATTCAGGCCTTCTCA	AGTTCCCCATGTGAAGAGCA
Control4 (C4)	GAGGAGGGTTTGAGCTCACT	CCTCTGTGTCTCCTGGTCAG
Control5 (C5)	TTCAGCCTTGGGTTCATTGC	TGGCTCACAGTAGGCTTTCA
Peak4 (P4)	GAGCTGGAAATGCACACCTC	ACCCCACCTTTGACATGTGA
Control6 (C6)	CTCTGTGTCTCCAAGGTCGT	TGCTCCAACATTTCAGTGGC
Control7 (C7)	TTACAGCTGCCACCCTTACA	ATGCCCCTGGTAGATTCGAC
Peak5 (P5)	CCCAGAGCAGAATGTTTGCA	CTGTCCCTGTCCCAGTAGTG
Peak6 (P6)	AGCACGTGACTATCCCAAGG	ATCTGCCCAAGGTCACAAAG

Supplementary Table 3 (continued).

NGS barcode primer (forward, barcode sequences in blue)

Name	Sequence (5'-3')
D501 long	AATGATACGGCGACCACCGAGATCTACACTATAGCCTACACTCTTTCCCTACACGACGCT
D502 long	AATGATACGGCGACCACCGAGATCTACACATAGAGGCACACTCTTTCCCTACACGACGCT
D503 long	AATGATACGGCGACCACCGAGATCTACACCCTATCCTACACTCTTTCCCTACACGACGCT
D504 long	AATGATACGGCGACCACCGAGATCTACACGGCTCTGAACACTCTTTCCCTACACGACGCT
D505 long	AATGATACGGCGACCACCGAGATCTACACAGGCGAAGACACTCTTTCCCTACACGACGCT
D506 long	AATGATACGGCGACCACCGAGATCTACACTAATCTTAACACTCTTTCCCTACACGACGCT
D507 long	AATGATACGGCGACCACCGAGATCTACACCAGGACGTACACTCTTTCCCTACACGACGCT
D508 long	AATGATACGGCGACCACCGAGATCTACACGTACTGACACACTCTTTCCCTACACGACGCT

NGS barcode primer (backward, barcode sequences in blue)

Name	Sequence (5'-3')
D701 long	CAAGCAGAAGACGGCATACGAGATCGAGTAATGTGACTGGAGTTCAGACGTGTGCT
D702 long	CAAGCAGAAGACGGCATACGAGATTCTCCGGAGTGACTGGAGTTCAGACGTGTGCT
D703 long	CAAGCAGAAGACGGCATACGAGATAATGAGCGGTGACTGGAGTTCAGACGTGTGCT
D704 long	CAAGCAGAAGACGGCATACGAGATGGAATCTCGTGACTGGAGTTCAGACGTGTGCT
D705 long	CAAGCAGAAGACGGCATACGAGATTTCTGAATGTGACTGGAGTTCAGACGTGTGCT
D706 long	CAAGCAGAAGACGGCATACGAGATACGAATTCGTGACTGGAGTTCAGACGTGTGCT
D707 long	CAAGCAGAAGACGGCATACGAGATAGCTTCAGGTGACTGGAGTTCAGACGTGTGCT
D708 long	CAAGCAGAAGACGGCATACGAGATGCGCATTAGTGACTGGAGTTCAGACGTGTGCT
D709 long	CAAGCAGAAGACGGCATACGAGATCATAGCCGGTGACTGGAGTTCAGACGTGTGCT
D710 long	CAAGCAGAAGACGGCATACGAGATTTCGCGGAGTGACTGGAGTTCAGACGTGTGCT
D711 long	CAAGCAGAAGACGGCATACGAGATGCGCGAGAGTGACTGGAGTTCAGACGTGTGCT
D712 long	CAAGCAGAAGACGGCATACGAGATCTATCGCTGTGACTGGAGTTCAGACGTGTGCT

Oligos for cloning sgRNA against MITF binding site P3

Name	Sequence (5'-3')
top strand	CACCGGATGCAGCCACTGTGCACG
bottom strand	AAACCGTGCACAGTGGCTGCATCC

siRNA sequences used in this study

Gene	Dharmacon cat#	siRNA sequence
MITF#1	D-008674-01	GAACGAAGAAGAAGAUUUA
MITF#2	D-008674-02	GCAGAUGGAUGAUGUAAUC
MITF#3	D-008674-03	GACCUAACCUGUACAACAA
MITF#4	D-008674-04	AGACGGAGCACACUUGUUA
JUN#1	D-003268-05	UGGAAACGACCUUCUAUGA
JUN#2	D-003268-08	UAACGCAGCAGUUGCAAAC
JUN#3	D-003268-09	GAGCGGACCUUAUGGCUAC
JUN#4	D-003268-22	AAGUCAUCAACCACGUUAA
